# Synthesis of novel 11*H*-indeno[1,2-*b*]quinoxaline derivatives and their in vitro and in silico insights into anticancer activities

**DOI:** 10.1038/s41598-026-64175-7

**Published:** 2026-08-12

**Authors:** Fatma A. El-Samahy, Ghada A. Eldeken, Ehab M. Zayed, Fayez H. Osman, Khaled Mahmoud, Galal E. H. Elgemeie

**Affiliations:** 1 Department of Green Chemistry, Chemical Industries Research Institute, National Research Centre, 33 El‑Buhouth St, Dokki, Giza, 12622 PO Egypt; 2 Pharmacognosy Department, National Research Centre, 33 El‑Buhouth St, Dokki, Giza, 12622 PO Egypt; 3 Chemistry Department, Faculty of Science, Capital University, Helwan, Cairo Egypt

**Keywords:** 11-Hydrazineylidene-11*H*-indeno[1,2-*b*]quinoxaline, Phosphonates, Antitumor activity, Molecular docking, SwissADME studies, Biochemistry, Cancer, Chemical biology, Chemistry, Computational biology and bioinformatics, Drug discovery

## Abstract

In the present work a novel series of 11*H*-indeno[1,2-*b*]quinoxaline derivatives were rationally designed and synthesised, with the aim of exploring their potential anticancer properties.

The novel compounds were studied by IR, ^1^H NMR, ^13^C NMR, ^31^P NMR, mass spectrometry, and single-crystal analysis. The anticancer efficacy of compounds **8a** and **8b** against the MCF-7 cell line demonstrated promising activity with IC50 values of 18.9 and 33.0 µg/mL, respectively. On the other hand, the antitumor efficacy of compounds **12** and **14** against the HCT116 cell line demonstrated encouraging results, with IC50 values of 47.3 and 21.4 µg/mL, respectively. Furthermore, in silico methodologies including ADME, BOILED-Egg, and bioactivity radar were employed to assess the oral bioavailability of the synthesized hybrids. Molecular docking was conducted to determine the docking poses and binding interactions of the derivatives with proteins bearing PDB: 6vj3 and 6GUE.

**Supplementary Information:**

The online version contains supplementary material available at 10.1038/s41598-026-64175-7.

## Introduction

**Fig. 1:**
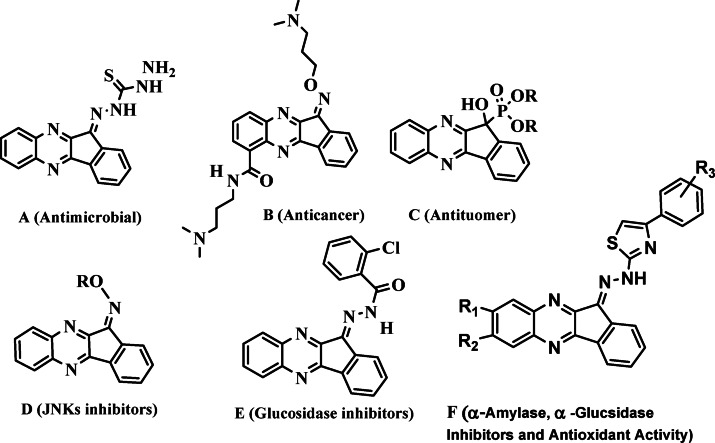
Some biologically active indeno[1,2-*b*]quinoxaline derivatives (**A**–**F**).

Due to their important role in drug development and substantial influence on the pharmaceutical industry, nitrogen-containing heterocyclic compounds have a prominent position among organic molecules^1^. Indenoquinoxaline specifically has been found to exhibit diverse biological properties and therapeutic value, such as antimicrobial activity (A)^2^, anticancer activity **(B**,** C)**^3–5^, *c*-Jun N-terminal kinase (JNK) inhibition **(D)**^6,7^, *α*- glucosidase inhibition **(E)**^8^, *α*-amylase, *α*-glycosidase inhibitors and antioxidant activity **(F)**^9,10^(Fig. 1).

Moreover, Hydrazones are a type of chemical molecule having the formula R1R2C=NNH_2_^11^, which is linked to ketones and aldehydes. Numerous biological and pharmacological characteristics, including antibacterial^12^, anti-inflammatory^13^, analgesic^14^, antifungal^15^, anti-tubercular^16^, antiviral^17,18^ and anticancer effects^19,20^ are possessed by these substances. When hydrazones are combined with other functional groups, compounds with distinct chemical and physical characteristics are produced. One of the most important groups is the acetyl group. The structure of the acyl hydrazones gives them considerable chemical and pharmacological malleability. Consequently, it’s a suitable choice for creating novel, physiologically active pharmacological compounds^21–26^.

Aldehydes or ketones are combined with hydrazides to form acylhydrazones with pharmacophore –CO–NH–N= group which are recognized for their antibacterial^27–30^, antiprotozoal^31–35^, antiviral^36^, analgesic and anti-inflammatory properties^37^.

Furthermore, it is commonly recognized that N-acyl hydrazones have a variety of biological characteristics as antitumoral^38^. For example, it was discovered that N-acyl hydrazones made from 3-hydroxy-2-naphthoic acid exhibited strong anticancer properties against the HepG2 and 769-P cell lines^39^.

A different investigation by Gautam et al.^40^ found that the prepared dipyrromethene N-acyl hydrazone derivatives showed a broad range of cytotoxic effects, from moderate to pronounced, against HL-60 and HCT-116 cancer cells.

Also, there have been numerous successful applications of the “magic” methyl idea in medicinal chemistry^41^. Methylation in late-stage functionalization has recently been used as a useful drug discovery method^42^.

The significance of organophosphorus compounds (OPCs), a broad family of organic chemicals with numerous uses, has increased gradually in recent years. There are parallels and variances among these compounds in each category and family. These compounds have a wide range of characteristics and, consequently, a wide range of applications because of their enormous variety^43^. The production of pesticides and insecticides is their primary use^44–46^ also these compounds have high biological activity as antitumor^3^.

As mentioned, hydrazone has a strong functional group, so its reaction with a carbonyl group forms compounds with highly appealing properties which can be shown to have a significant role in pharmacological activities^47–51^.

This work involved the synthesis of *a* novel 11*H*-indeno[1,2-*b*]quinoxaline derivatives and the evaluation of their anticancer activities. Additionally, the in-silico docking studies of compounds (**8a**,** 8b**,** 12** and **14**) were shown by the ADME investigations.

## Results and discussions

### Chemistry

**Scheme 1:**
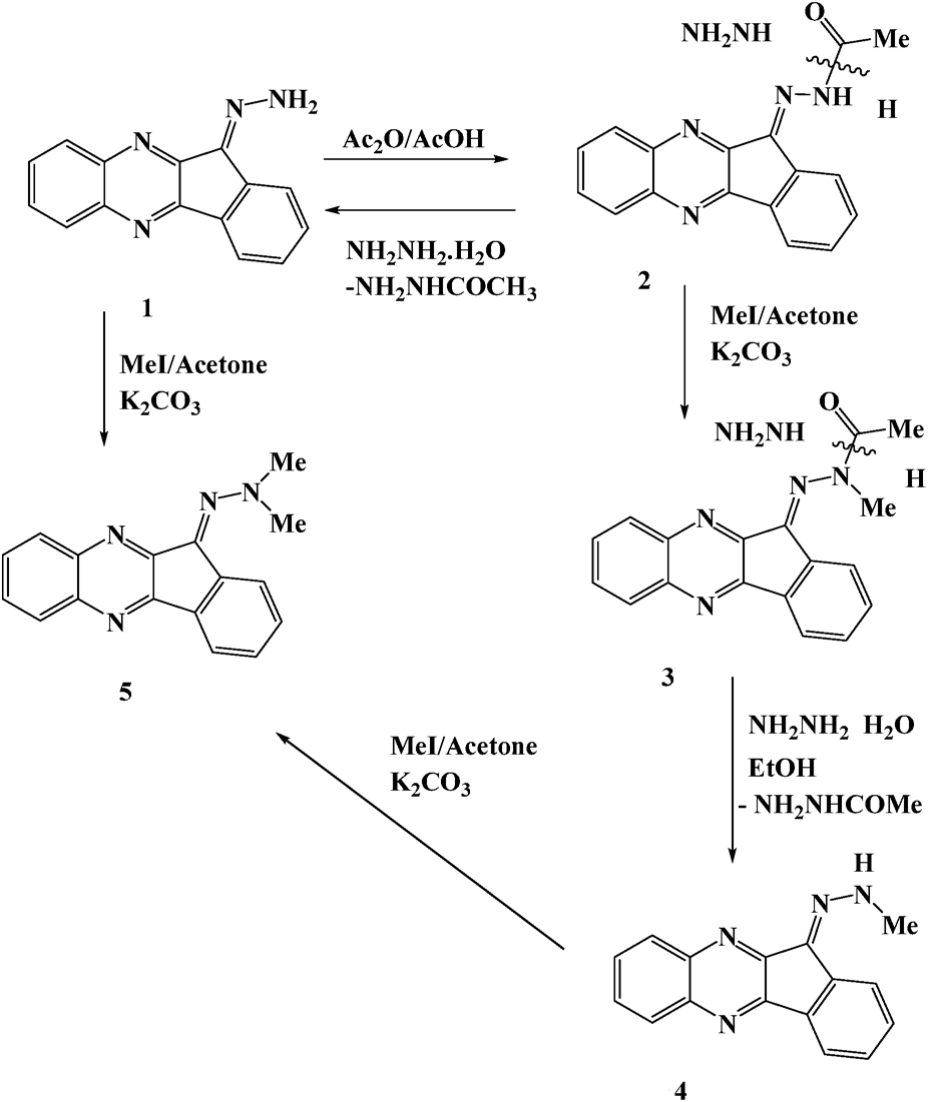
Synthesis of 11-(hydrazineylidene)-11*H*-indeno[1,2-*b*]quinoxaline derivatives **2–5.**

Acetylation of hydrazone **1** using acetic anhydride and acetic acid afforded monoacetylated derivative **2**^52^. Methylation of acylated compound **2** with methyl iodide in acetone and in the presence of anhydrous potassium carbonate gave the methylated *N’*-(11*H*-indeno[1,2-*b*]quinoxalin-11-ylidene)-*N*-methylacetohydrazide **(3)** as white crystals in good yields. The structure of **3** is conformed by ^1^H NMR which showed the presence of two methyl groups at δ = 2.44, 3.80 ppm (2 s, COCH_3_, NCH_3_), with the absence of NH group. On the other hand, the amides **2** and **3** were reacted with hydrazine hydrate in absolute ethanol to afford the adduct **1** and **4** through the elimination of acyl hydrazides^53^. Methylation of hydrazones **1** and **4** with methyl iodide in acetone in the presence of anhydrous potassium carbonate afforded di-methylated product **5** in good yield (Scheme 1).

**Scheme 2:**
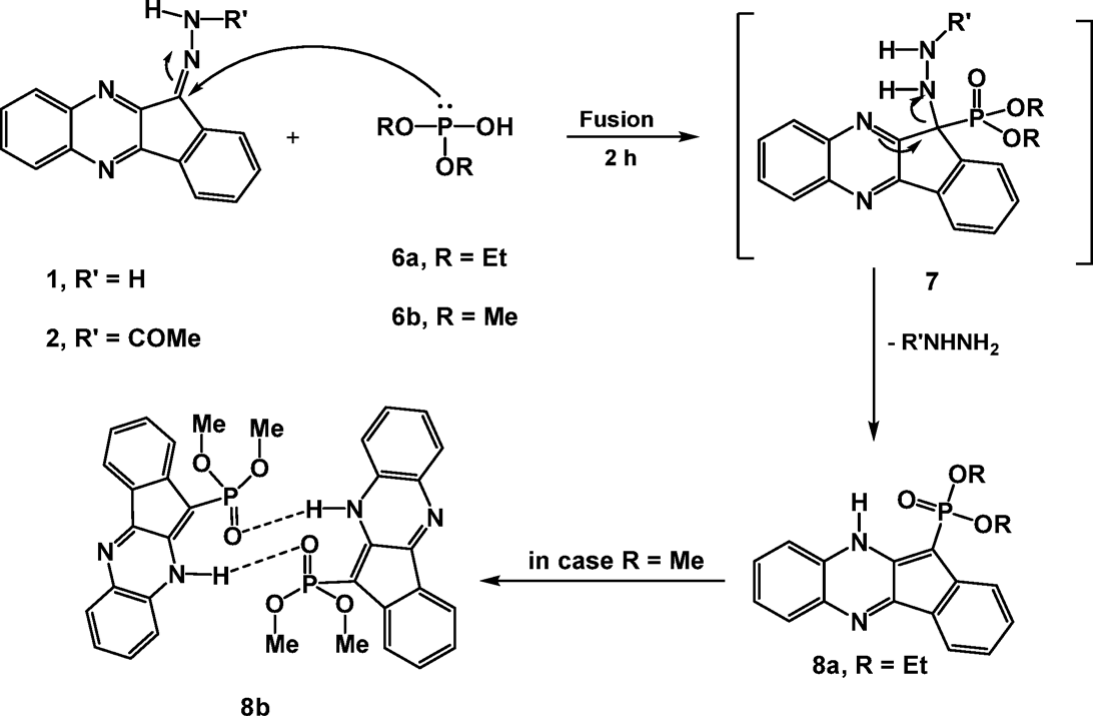
Synthesis of dialkyl (10*H*-indeno[1,2-*b*]quinoxalin-11-yl)phosphonates **(8a**,** b).**

Reaction of 11-hydrazineylidene-11*H*-indeno[1,2-*b*]quinoxaline **(1)** and/or *N’*-(11*H*-indeno- [1,2-*b*]quinoxalin-11-ylidene)acetohydrazide **(2)** with dialkyl phosphites **(6a**,** b)** was heated without solvent for about 2 h led to the synthesis of unexpected diethyl (10*H*-indeno[1,2-*b*]quinoxalin-11-yl)phosphonate **(8a)** and dimethyl (10*H*-indeno[1,2-*b*]quinoxalin-11-yl)phosphonate **(8b)** in good yield. An illustrative mechanism for the formation of compound **8** is proposed in Scheme 2 shows the nucleophilic attack by the phosphite-phosphorus on the azine C = N bond attached to the indenoquinoxaline moiety in **1** and **2** to form the intermediate **7**, which then undergoes the elimination of hydrazine derivatives with formation of the phosphonates **8.** The ^1^H NMR spectra of **8a** as an example indicated the presence of two ethoxy groups at *δ* 1.19, 1.21 [2 t, *J*_*HH*_ = 7.0 Hz, P(OCH_2_**CH**_**3**_)_2_], 3.95, 3.96 [2 q, *J*_*HH*_ = 7.0 Hz, P(O**CH**_**2**_CH_3_)_2_] ppm and its ^31^P NMR spectra showed the signal at *δ* 20.95 ppm (Scheme 2).

In order to establish the structure of dialkyl (10*H*-indeno[1,2-*b*]quinoxalin-11-yl)phosphonates **(8a**,** b)** unambiguously, the X-ray crystal structures of **8a**,** b** were determined (Fig. 3)^54^.

**Fig. 2:**
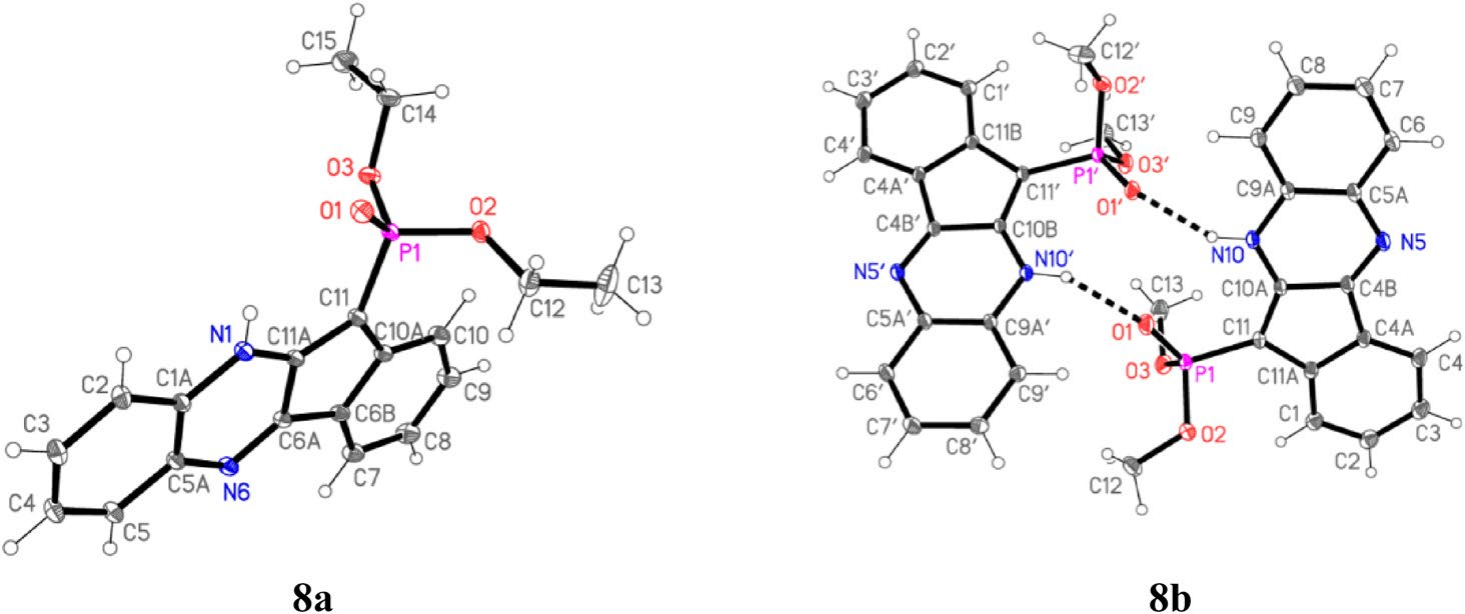
ORTEP (single crystal X-ray diffraction analysis) of **8a**,** b**^54^.

**Scheme 3:**
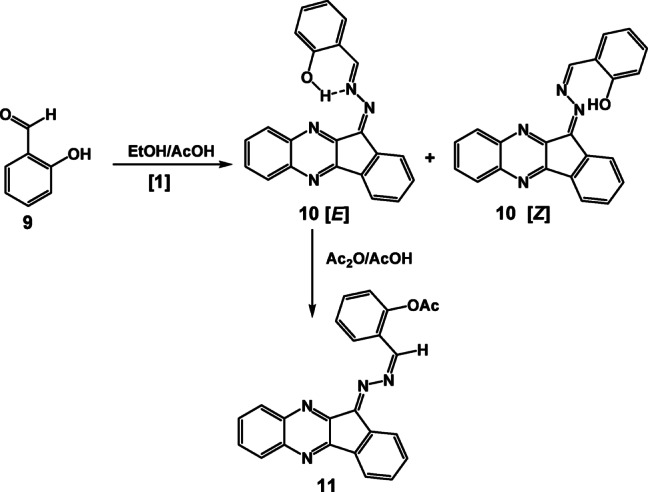
Synthesis of indenoquinoxaline derivatives **10 E**,** Z** and **11.**

The condensation of 2-hydroxybenzaldehyde **(9)** with the amino group of 11-hydrazineyli-dene-11*H*-indeno[1,2-*b*]quinoxaline **(1)** in absolute ethanol and in the presence of acetic acid afforded 2-((*E*)-(((*Z*)-11*H*-indeno[1,2-*b*]quinoxalin-11ylidene)hydrazineylidene)methyl)phenol **(10*E*)** and 2-((*Z*)-(((*Z*)-11*H*-indeno[1,2-*b*]quinoxalin-11ylidene)hydrazineylidene)methyl)- phenol **(10*Z*)**. We couldn’t isolate the compound **10*Z*** in pure form with column and plate chromatography which identified with ^1^H NMR. Next, the compound 2-((*E*)-(((*Z*)-11*H*-indeno[1,2-*b*]quinoxalin-11-ylidene)hydrazineylidene)methyl)phenyl acetate **(11)** was formed in a good yield from the reaction of **10** ***E*** with acetic anhydride in acetic acid (Scheme 3).

**Scheme 4:**
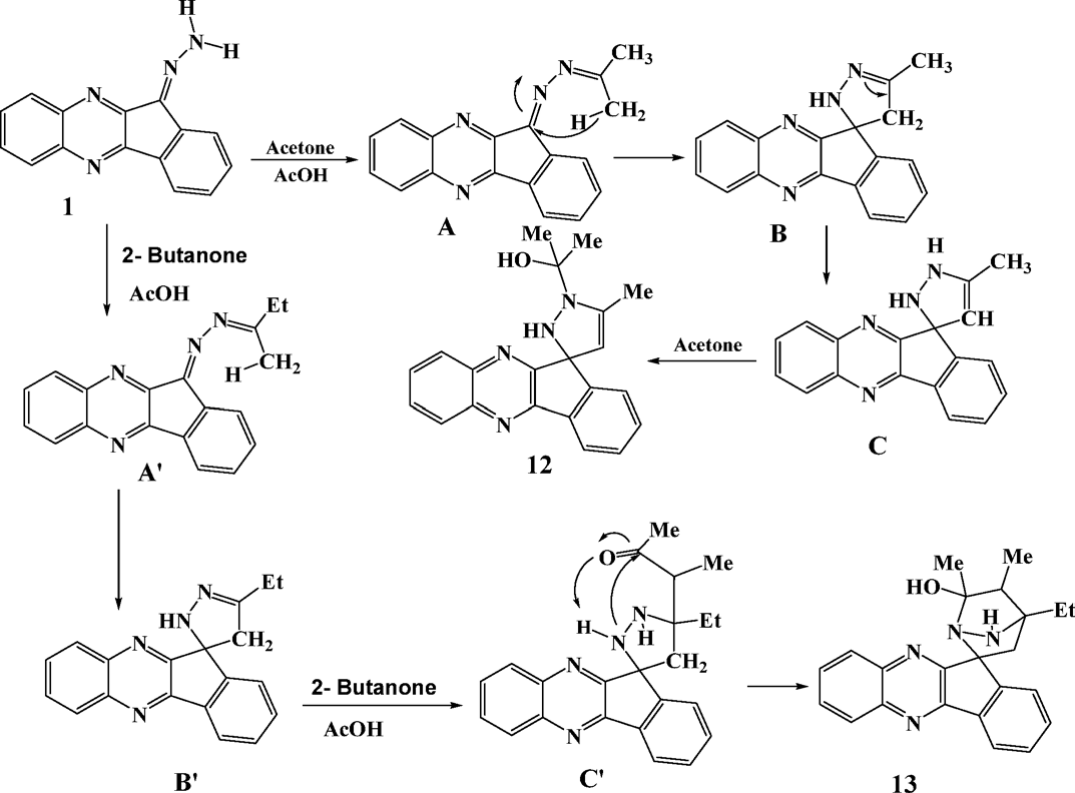
The reaction of 11-hydrazineylidene-11*H*-indeno[1,2-*b*]quinoxaline with acetone and 2-butanone in the presence of acetic acid.

Nucleophilic reaction of indenoquinoxaline hydrazone **(1)** with acetone and 2-butanone with a few drops of acetic acid at about 50–70 °C gave the corresponding products 2-(5’-Methylspiro[indeno[1,2-*b*]quinoxaline-11,3’-pyrazol]-1‘(2’H)-yl)propan-2-ol **(12)** and 4-ethyl-5,6-dimethyl-1,7-diazaspiro[bicyclo[2.2.1]heptane-2,11’-indeno[1,2-b]quinoxalin]-6-ol (**13)** in a good yields (Scheme 4). The suggested reaction pathway proceeds via nucleophilic attack of the primary amine on the ketone carbonyl group, forming the N-substituted imine **A**, **A`**. The resulting Schiff base intermediate **A**,** A`** undergoes intramolecular cyclization to give **B**, **B`** which reacted with second molecule of ketone through Michael addition intermediate **C**, **C`** in order to yield **12** and **13**, respectively. The structures of **12** and **13** were confirmed by the spectral analyses detailed in the experimental section. The absorptions of OH and NH were recorded in the IR spectrum of **12** at 3361 and 3152 cm^− 1^, respectively. In ^1^H NMR spectrum of **12**, the protons of methyl groups were observed as singlet at δ 1.58 (2Me) and 2.18 (Me) ppm. The molecular ion peak was observed in the mass spectrum at m/z = 344, which confirmed the molecular formula C_21_H_20_N_4_O.

**Scheme 5:**
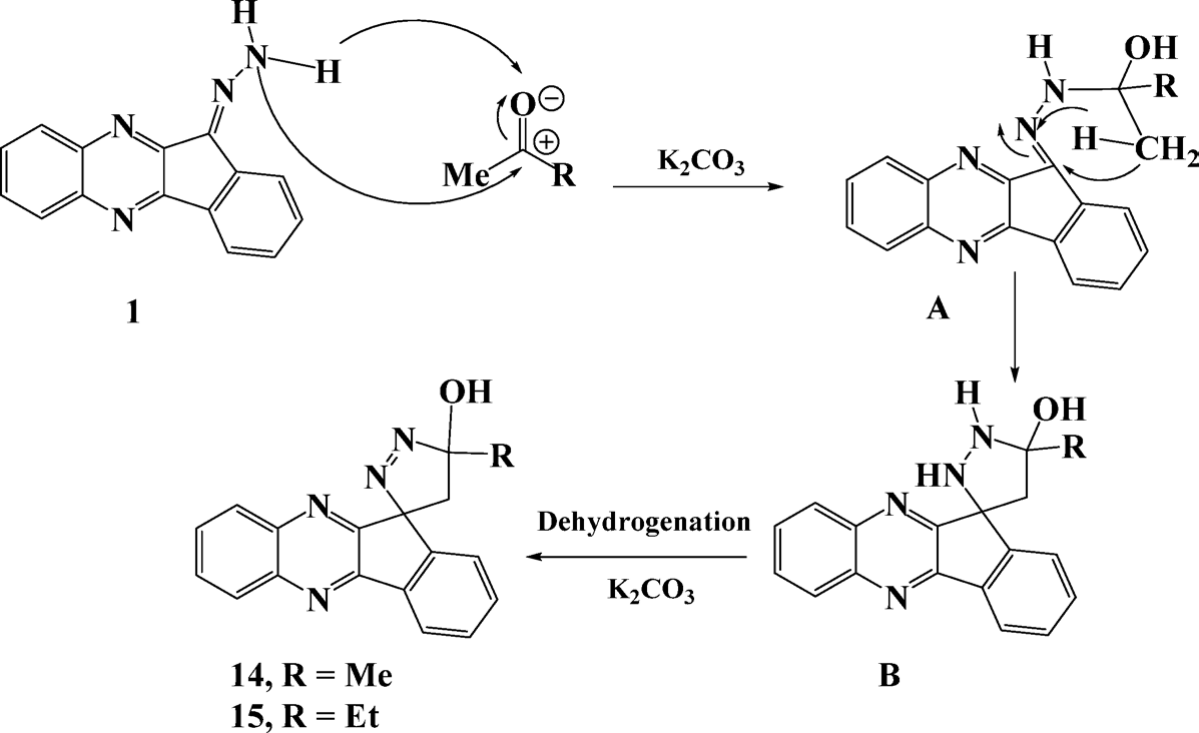
The reaction of 11-hydrazineylidene-11*H*-indeno[1,2-*b*]quinoxaline with acetone and 2-butanone in presence of potassium carbonate.

On the other hand, repeating the above reaction of **1** with ketone using potassium carbonate afforded 5’-methyl-4’,5’-dihydrospiro[indeno[1,2-*b*]quinoxaline-11,3’-pyrazol]-5’-ol **(14)** and 5’-ethyl-4’,5’-dihydrospiro[indeno[1,2-*b*]quinoxaline-11,3’-pyrazol]-5’-ol **(15)**. Nucleophilic attack of amino nitrogen on the carbonyl carbon of ketone to give the enolate structure **A**, which undergoes the cyclization through nucleophilic addition on the imine group to give intermediate **B**. Dehydrogenation of **B** in presence of potassium carbonate led to the formation of the pyrazole derivatives **14** and **15.** The structure of the compounds **14** and **15** was confirmed. The IR spectra of compounds **14** and **15** exhibited the presence of OH peak at 3361 cm^− 1^. The ^1^H NMR spectrum of **14** showed signals at δ 2.15 (s, Me) and two doublet signals at δ 3.24, 3.94 (*J*_*HH*_ = 17 Hz, CH_2_) ppm, additionally, singlet signals at δ 5.19 ppm attributable to OH. Moreover, the mass spectra of **14** revealed [M^+^, 23%] ion peak at *m/z* 302 (Scheme 5).

The mixture of compound **1** and urea in acetic acid was refluxed at 80 °C for about 2 h to produce N’-(11*H*-indeno[1,2-*b*]quinoxalin-11-ylidene)acetohydrazide **(2).** Carrying out the same reaction in acetic acid without presence of urea, compound **2** was obtained after 4 h with 80% yield.

### Cytotoxicity assay

A literature search indicated that 11*H*-indeno[1,2-*b*]quinoxalin-11-one **(1)** have chemotherapeutic efficacy^6–10^. This prompted us to evaluate their inhibitory efficacy against tumour cell lines, leading us to seek a scientific alternative with various substituents within the 11*H*-indeno[1,2-*b*]quinoxalin-11-one molecule. Ten indenoquinoxaline derivatives **(3**,** 4**,** 8a**,** 8b**,** 10E**,** 11**,** 12**,** 13**,** 14** and **15)** have been evaluated in vitro for their cytotoxic effects against the human colon cell line (HCT116) and human Caucasian breast cancer (MCF7). The most effective activity exhibited IC_50_ values of 18.9 µg/mL **(8a)** and 33.0 µg/mL **(8b)** against MCF-7 cells. On the other hand, compounds **12** (47.3 µg/mL), **14** (21.4 µg/mL) and **8a** (52.8 µg/mL) have most promising cytotoxicity activity on human colon cell line (HCT116) Table 1.

The phosphonates exhibited significant cytotoxic action against MCF7 human Caucasian breast cancer cells adenocarcinoma, although the interest was in moderate activities exerted on colon cell line (HCT116) with 52.2% **(8a)** and 72.9% **(8b)** inhibition in cell growth, respectively. The results of cytotoxic activity of the 11*H*-indeno[1,2-*b*]quinoxalin-11-one derivatives are shown in Table 1. Generally, the results indicate that the introduction of phosphorus groups into11*H*-indeno[1,2-*b*]quinoxalin-11-one ring increases the cytotoxic activity and furthermore propose that the diethyl (10*H*-indeno [1,2-*b*]quinoxalin-11-yl)phosphonate **(8a)** displays the maximum promising activity. Compounds **10*E***, **11** and **13** appeared to be inactive towards human Caucasian breast adenocarcinoma (MCF7) and human colon cell line (HCT116). The IC50 value was calculated based on the concentration at which 50% of cell survival was observed. Dimethyl sulfoxide (DMSO) served as a negative control.

**Table 1:** Cytotoxicity of the synthesized 11 H-indeno [1,2-b]quinoxalin-11-one derivatives towards human Caucasian breast cancer (MCF7) and human HCT116 [Colon cell line] with µg/mL.

Sample code	IC_50_ of MCF-7 cells (μg/mL)	IC_50_ of HCT116 Cells (μg/mL)	Sample code	IC_50_ of MCF-7 cells (μg/mL)	IC_50_ of (HCT116 cells (μg/mL)
3	60.0±1.5	ND^[a]^	12	58.6±2.3	47.3±1.4
4	70.2±1.6	57.2±0.8	13	ND	ND
8a	18.9±1.2	52.8±0.9	14	65±1.7	21.4±0.9
8b	33.0±1.2	72.9±0.6	15	60.9±0.3	57±0.8
10 *E*	ND	ND	DMSO	ND	ND
11	ND	ND	Negative control	ND	ND
+ve control Doxorubicin	45.02±1.3	65.1±0.9			

[a] ND, not detected ± S.D.

### Expression of *BCl2*, *BAX* and *P53* in MCF-7 cell lines

**Fig. 3:**
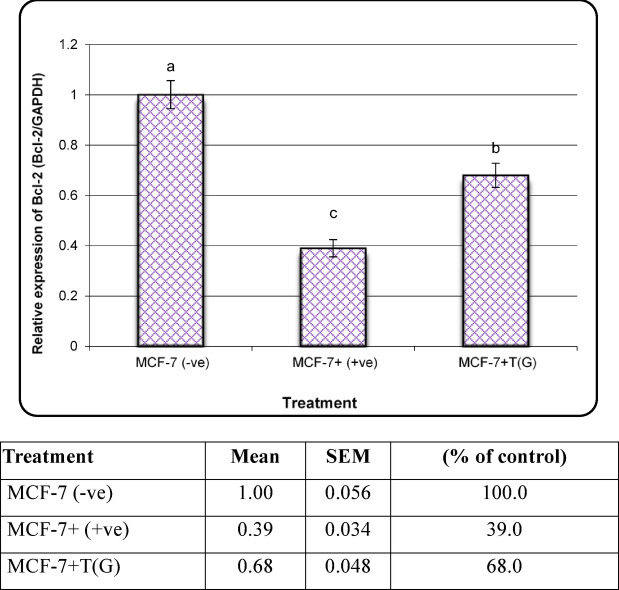
The alterations of *Bcl-2* gene expression in negative and treated MCF-7 cell lines. Data are presented as mean ± SEM. ^a, b,c^: Mean values within tissue with unlike superscript letters were significantly different (*P* < 0.05).

**Fig. 4:**
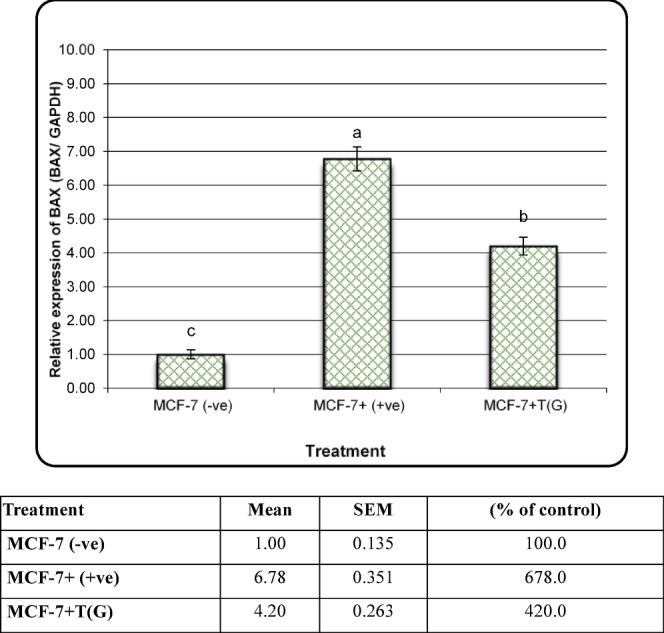
The alterations of *BAX* gene expression in negative and treated MCF-7 cell lines. Data are presented as mean ± SEM. ^a, b,c^: Mean values within tissue with unlike superscript letters were significantly different (*P* < 0.05).

**Fig. 5:**
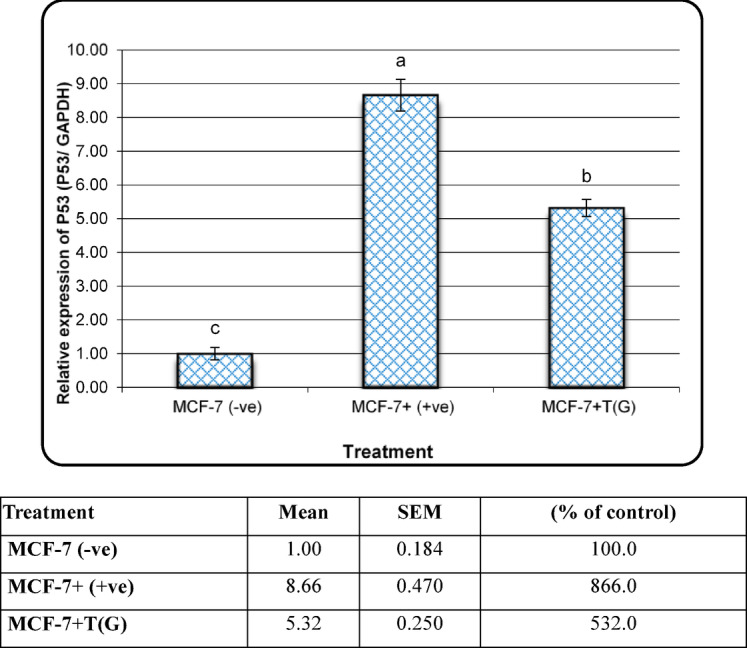
The alterations of *P53* gene expression in negative and treated MCF-7 cell lines. Data are presented as mean ± SEM. ^a, b,c^: Mean values within tissue with unlike superscript letters were significantly different (*P* < 0.05).

Expreesion analysis of *Bcl-2*, *BAX* and *P53* genes in breast cancer cell lines (MCF-7) are represented in Figs. 3, 4 and 5.

The results showed that the expression levels of *Bcl-2* gene (B-cell lymphoma 2) as anti-apoptotic gene that promotes cell survival were up-regulated (1.00 ± 0.056) singnificantly (*P* < 0.05) in negative samples of breast cancer cell lines (MCF-7) compared with treated cell lines (Fig. 3). However, the expression levels of *Bcl-2* gene in MCF-7 treated with Doxo as reference drug were decreased (0.39 ± 0.034) significantly (*P* < 0.05) by 39% as compared with negative MCF-7 (1.00 ± 0.056) (Fig. 3). Additionally, the expression levels of *Bcl-2* gene in MCF-7 treated with T(G) were decreased (0.68 ± 0.048) significantly (*P* < 0.05) by 68.0% as compared with negative MCF-7 (1.00 ± 0.056) (Fig. 3).

The results showed that the expression levels of *BAX* gene (Bcl-2-associated X protein) as pro-apoptotic gene that promotes apoptosis were down-regulated (1.00 ± 0.135) singnificantly (*P* < 0.05) in negative samples of human breast cancer cell lines (MCF-7) compared with treated MCF-7 cell lines (Fig. 4). However, the expression levels of *BAX* gene in MCF-7 treated with Doxo as reference drug were increased (6.78 ± 0.351) significantly (*P* < 0.001) by 678.0% as compared with negative MCF-7 (1.00 ± 0.135) (Fig. 4). Moreover, the expression levels of *BAX* gene in MCF-7 treated with T(G) were reduced (4.20 ± 0.263) significantly (*P* < 0.01) by 420.0% as compared with negative MCF-7 (1.00 ± 0.135) (Fig. 4).

The results showed that the expression levels of *P53* gene as crucial tumor suppressor involved in apoptotic cell death were down-regulated (1.00 ± 0.184) singnificantly (*P* < 0.05) in negative samples of human breast cancer cell lines (MCF-7) compared with treated cell lines (Fig. 5). However, the expression levels of *P53* gene in MCF-7 treated with Doxo drug were increased (8.66 ± 0.470) significantly (*P* < 0.001) by 866.0% as compared with negative MCF-7 (1.00 ± 0.184) (Fig. 5). Additionally, the expression levels of *P53* gene in MCF-7 treated with T(G) were increased (5.32 ± 0.250) significantly (*P* < 0.01) by 532.0% as compared with negative MCF-7 (1.00 ± 0.184) (Fig. 5).

### Cell cycle analysis

Cell cycle analysis was carried out to detect the probable changes in the cell cycle phases in control and treated cells with 8a.

**Fig. 6:**
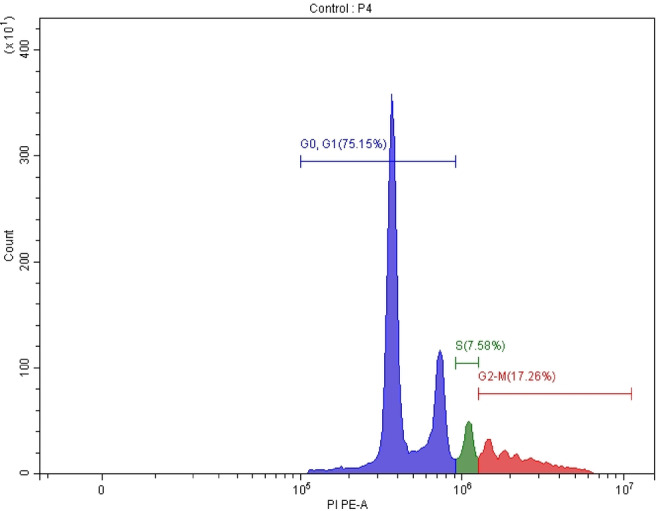
Cytometric analysis of the cell cycle distribution of cells with –ve control.

**Fig. 7:**
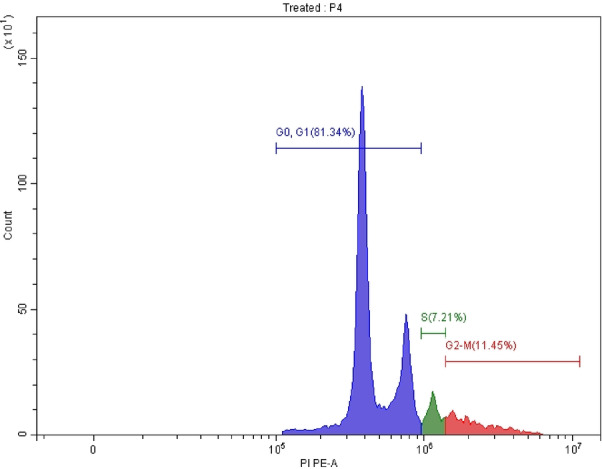
Cytometric analysis of the cell cycle distribution of cells with sample-treated..

**Fig. 8:**
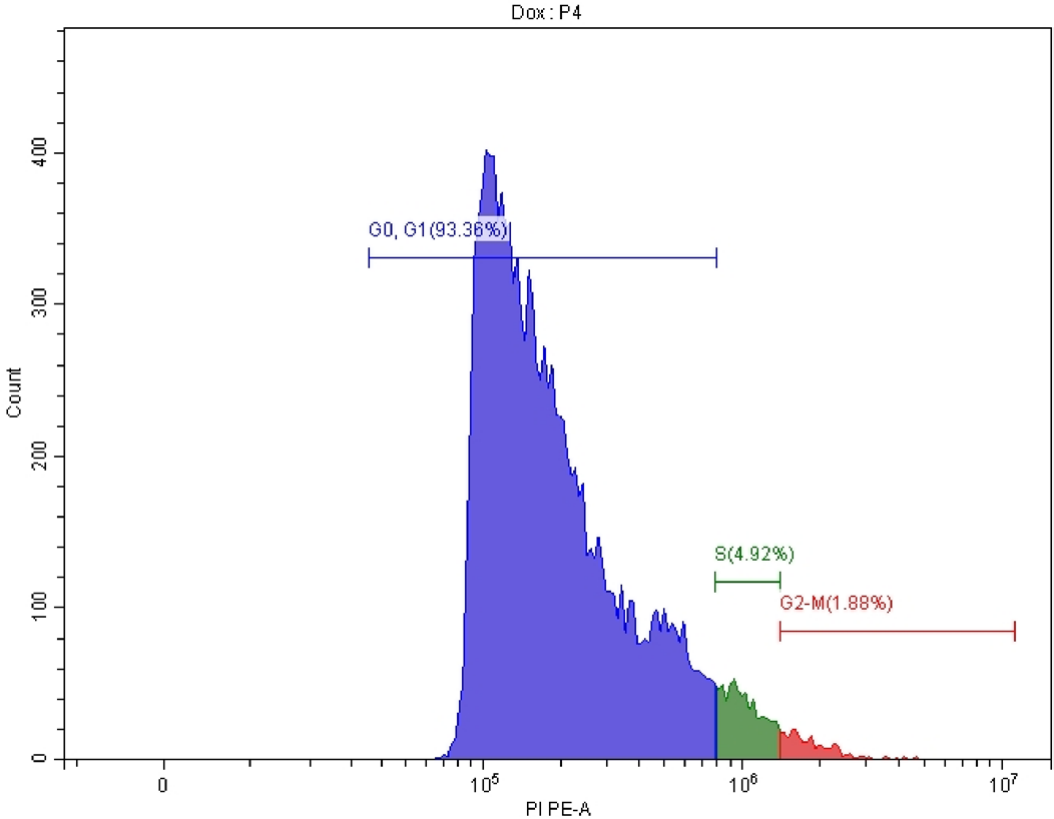
Cytometric analysis of the cell cycle distribution of cells with dox.

Flow cytometric analysis of the cell cycle distribution of cells treated with sample showed phase G0/G1 arrest from 75.15% (control, Fig. 6) to 81.34% (treated, Fig. 7). Flow cytometric analysis of the cell cycle distribution of cells treated with the positive control doxorubicin showed arrest at G0/G1 phase of from 75.15% (control, Fig. 6) to 93.36% (dox, Fig. 8).

### Molecular docking

The protein preparation was executed via PyMOL. The protein structures were sourced from the RCSB-PDB database and prepped for docking by eliminating water molecules.

**Fig. 9:**
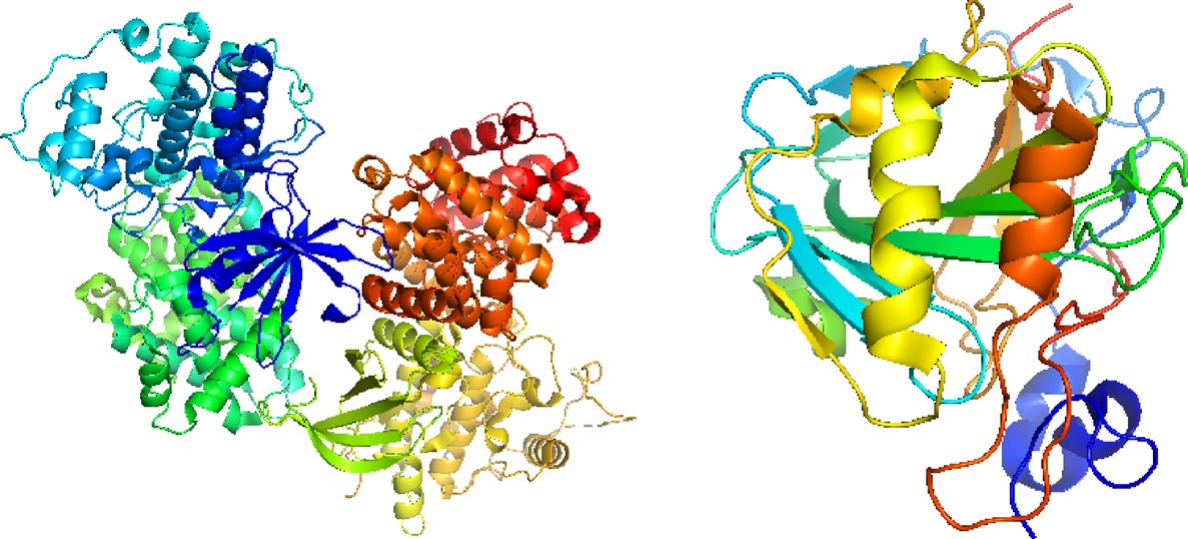
Crystal structure of proteins (PDB ID: 6GUE) and (PDB ID: 6VJ3).

These compounds were docked into several proteins linked to breast cancer (PDB ID: 6vj3) and colon cancer (PDB ID: 6GUE) for protein 6GUE water molecules and all protein chains except chain A were deleted prior to docking (Fig. 9).

The molecular docking study was performed to investigate the binding modes of the synthesized compounds within the active sites of the selected protein targets (PDB IDs: 6VJ3 and 6GUE). The docking approach was confirmed by re-docking of the co-crystallized ligands into the corresponding binding sites, yielding RMSD values of 1.4 Å for 6VJ3 and 0.77 Å for 6GUE, confirming the reliability and accuracy of the employed docking procedure.

The docking results indicated favourable binding affinities for several compounds toward both targets. For 6VJ3, compound 8a exhibited the most stable binding mode (− 9.4 kcal/mol), forming hydrogen bond interactions with ASN62 and THR199.

For 6GUE, compound 14 showed the best predicted binding affinity (− 8.8 kcal/mol), characterised by hydrogen bond interactions involving GLU12 and TYR15. Additional non-covalent interactions facilitated the stabilization of the ligand–protein complexes.

Additionally, the ligands exhibited several additional interactions. The associated docking scores are presented in the (Table 2).

The docking results are regarded as predictive computational support for potential ligand–protein interactions rather than direct biological evidence of activity.

Overall, these findings provide useful insights into the binding behaviour of the investigated compounds; however, further experimental validation is required to confirm their biological activity.

**Table 2:** Docking energy (kcal/mol) and binding interactions of the ligands docked into the active human MAPK8 proteins.

Compounds	Receptor	Conventional Hydrogen bond	Pi Donor Hydrogen bond	Pi-alkyl/Alkyl	Pi-Pi Tshaped / Pi-Pi T staacked	Pi- Sigma	Pi- cation /pi-anion	Binding energy (Kcal/mol)
**8a**	6vj3	ASN62(2.13Å)	THR199(3.36Å)	LEU198(4.7 Å) VAL143(6.06 Å) VAL121(5.2 Å,4.9 Å HIS96(4.45 Å) ALA65(3.59 Å)				-9.4
**8b**	6vj3	GLN92(2.36 Å) ASN67(2.18Å)		VAL121(4.78 Å) VAL143(4.79 Å) LEU198(3.8 Å) ALA65(4.7 Å)		LEU198(3.82 Å) THR200(3.80 Å)		-8.6
**12**	6vj3	PRO201 (2.42 Å)		VAL121 (4.69 Å)	PHE131 (4.90Å) HIS94 (5.25Å)	LEU198 (3.84 Å)		-8.2
**14**	6vj3	ASN 67(2.77 Å) ASN62(2.60 Å) (2.29 Å)		LEU198(5.01 Å)		HIS94(3.97 Å)		-8
**8a**	6GUE	LYS129(2.45Å) (2.57Å)	TYR15(3.22Å)	TYR15(4.76Å)		VAL18(3.92Å)	ASP86(3.75Å)	-7.2
**8b**	6GUE	TYR15(2.97 Å) LYS129(2.67Å)		VAL18(5.07 Å)		VAL 18(3.80 Å)	ASP 86(3.79 Å)	-7
**12**	6GUE	LYS 250 (5.33 Å) GLN 246 (5.16 Å)		ARG214 (5.74 Å) ARG217 (6.50 Å) LEU202 (5.82 Å)				-7.7
**14**	6GUE	GLU 12 (2.76 Å) TYR15 (1.85 Å)		VAL18 (4.99Å)		VAL 18 (3. 65 Å)	ASP86 (3.56 Å)	-8.8

**Fig. 10:**
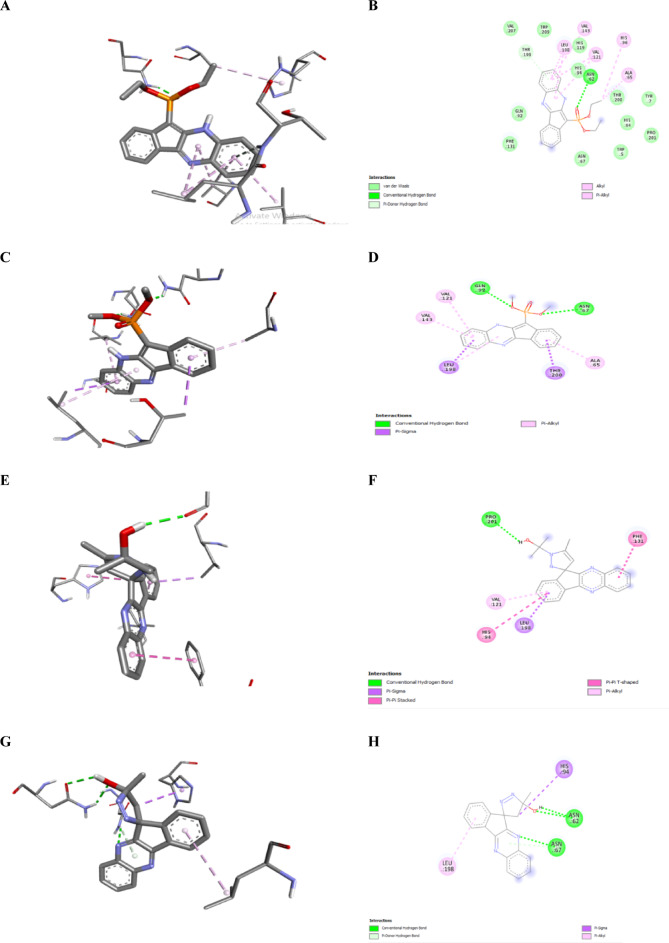
Molecular docking of compounds **8a** (**A**, **B**), **8b** (**C**, **D**), **12** (**E**, **F**) and **14** (**G**, **H**) with breast protein (6vj3) 3 D and 2 D.

**Fig. 11:**
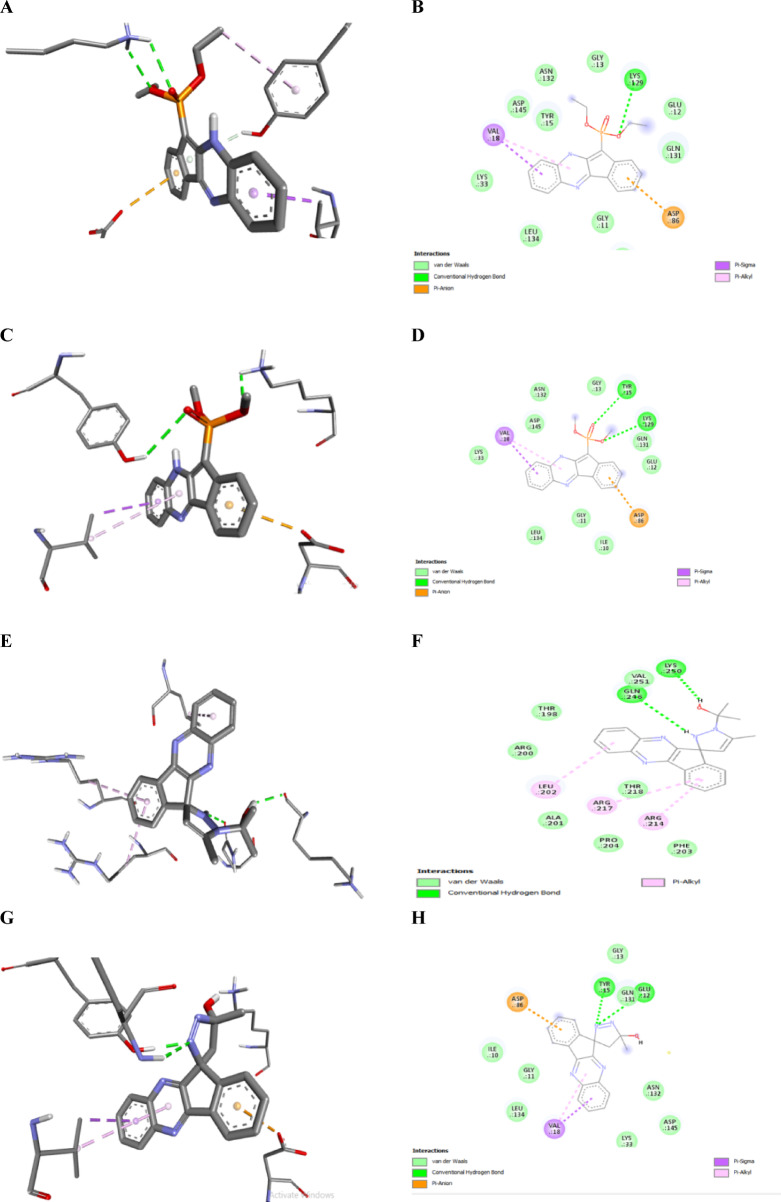
Molecular docking of compounds **8a** (**A**, **B**), **8b** (**C**, **D**), **12** (**E**, **F**) and **14** (**G**, **H**) with colon protein (6GUE).

**Fig. 12:**
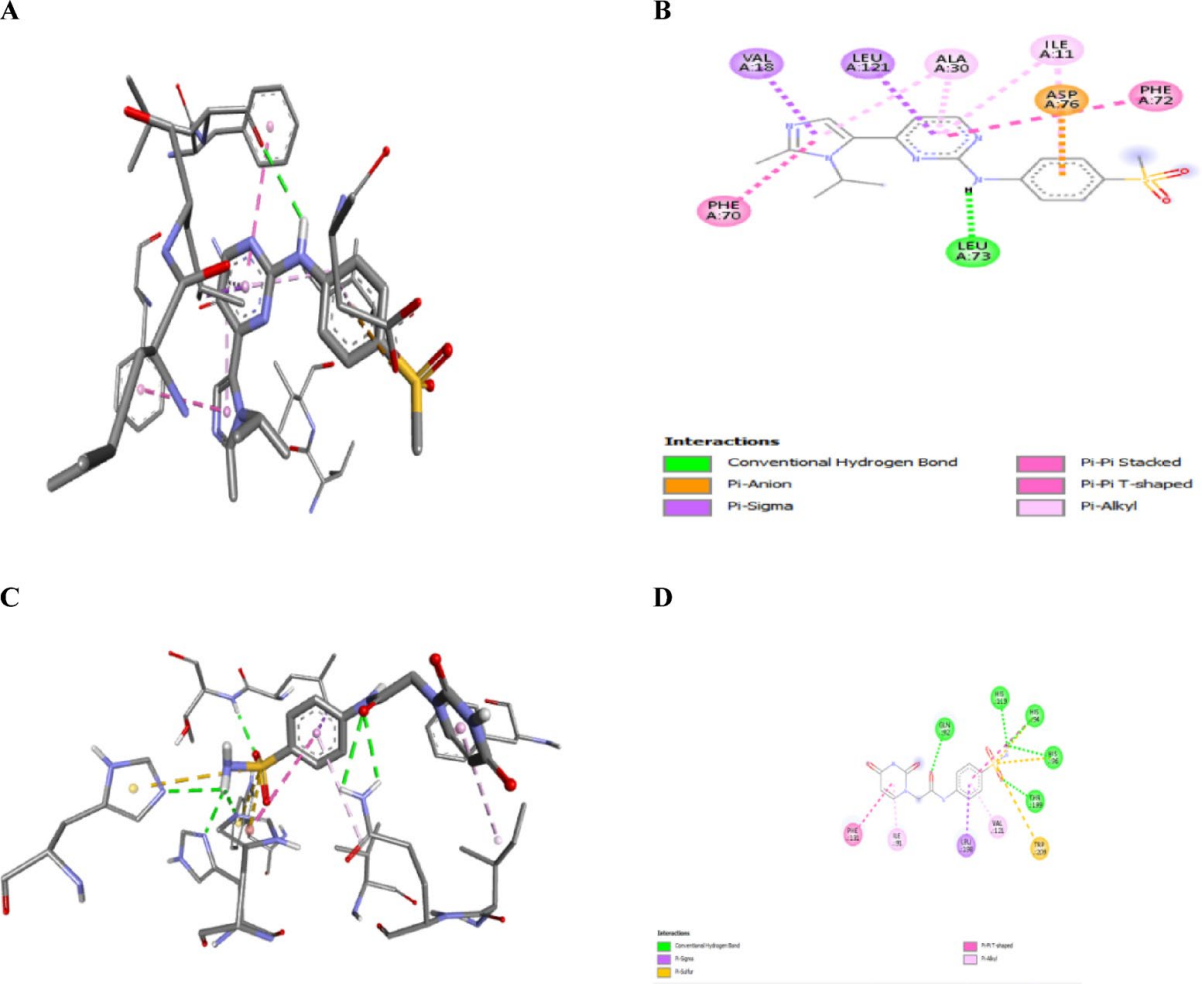
Molecular docking of co-crystallized 6GUE (**A**, **B**) and co-crystalized 6VJ3 (**C**, **D**).

**Fig. 13:**
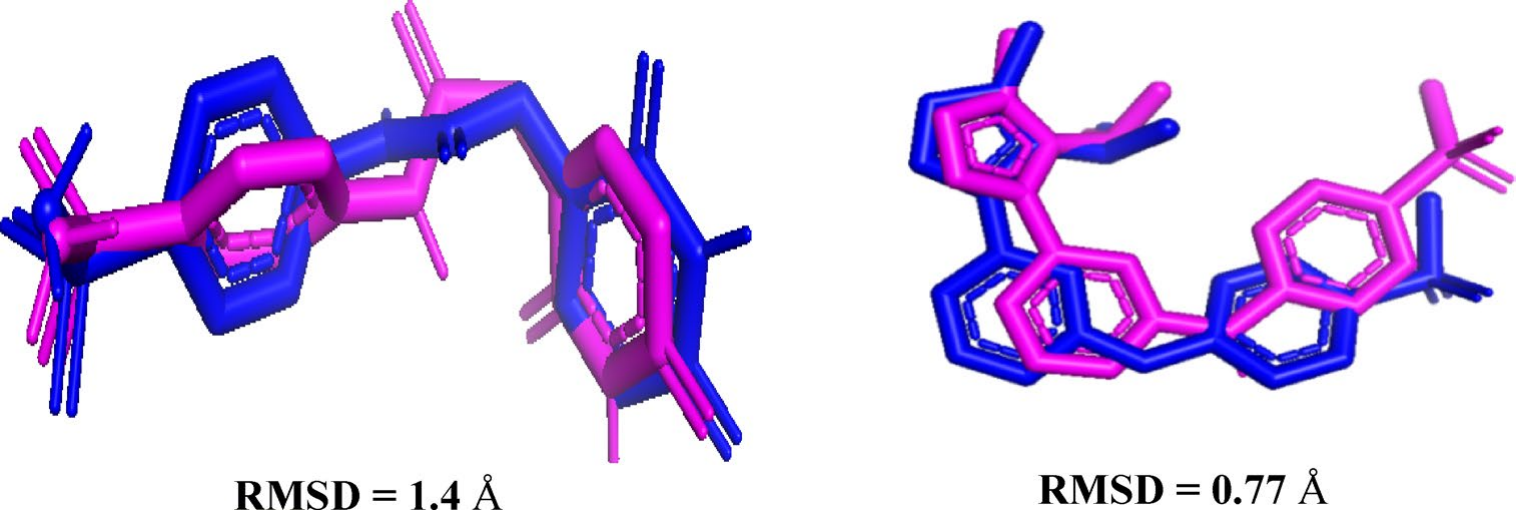
Superimposition of the re-docked (magenta) and co-crystallized (blue) reference ligand poses within the active sites PDB (6VJ3) and PDB (6GUE).

The 3D and 2D diagrams of compounds **8a**, **8b**, **12** and **14** and co-crystallized ligands with proteins breast (6vj3) and colon (6GUE) were showed in Figs. 10, 11 and 12. The rmsd of co-crystalized 6VJ3 and co-crystallized 6GUE was showed in Fig. 13.

### SwissADME analysis

The number of rotatable bonds in all compound’s ranges from 1 to 5, demonstrating that the molecule is flexible with respect to its biological target. Furthermore, all compounds possess fewer than 10 hydrogen bond acceptor (HBA) sites. They possess less than 5 hydrogen bond donors (HBD) sites. Topological polar surface area (TPSA) values for all medicines are below 140 Å², indicating great oral bioavailability.

According to the drug-likeness prediction, the synthesized compounds satisfy Lipinski’s rules for oral pharmaceuticals. All compounds have a molecular weight below 500 g/mol, Log P values below 5, with a maximum of 5 hydrogen bond donors (HBD) and no more than 10 hydrogen bond acceptors (HBA) Table 3.

**Table 3:** The physicochemical parameters of the compounds **8a**, **8b**, **12** and **14**.

Compounds	MW	Rotatable bonds	H-bond acceptors	H- bond donors	TPSA	(I LOG *P*)	Lipinski violation	Bioavailability Score
**8a**	354.34 g/mol	5	4	1	74.02 Å²	3.18	0	0.55
**8b**	326.29 g/mol	3	4	1	74.02 Å²	2.88	0	0.55
**12**	344.41 g/mol	1	4	2	61.28 Å²	2.99	0	0.55
**14**	302.33 g/mol	0	5	1	70.73Å²	2.64	0	0.55

**Fig. 14:**
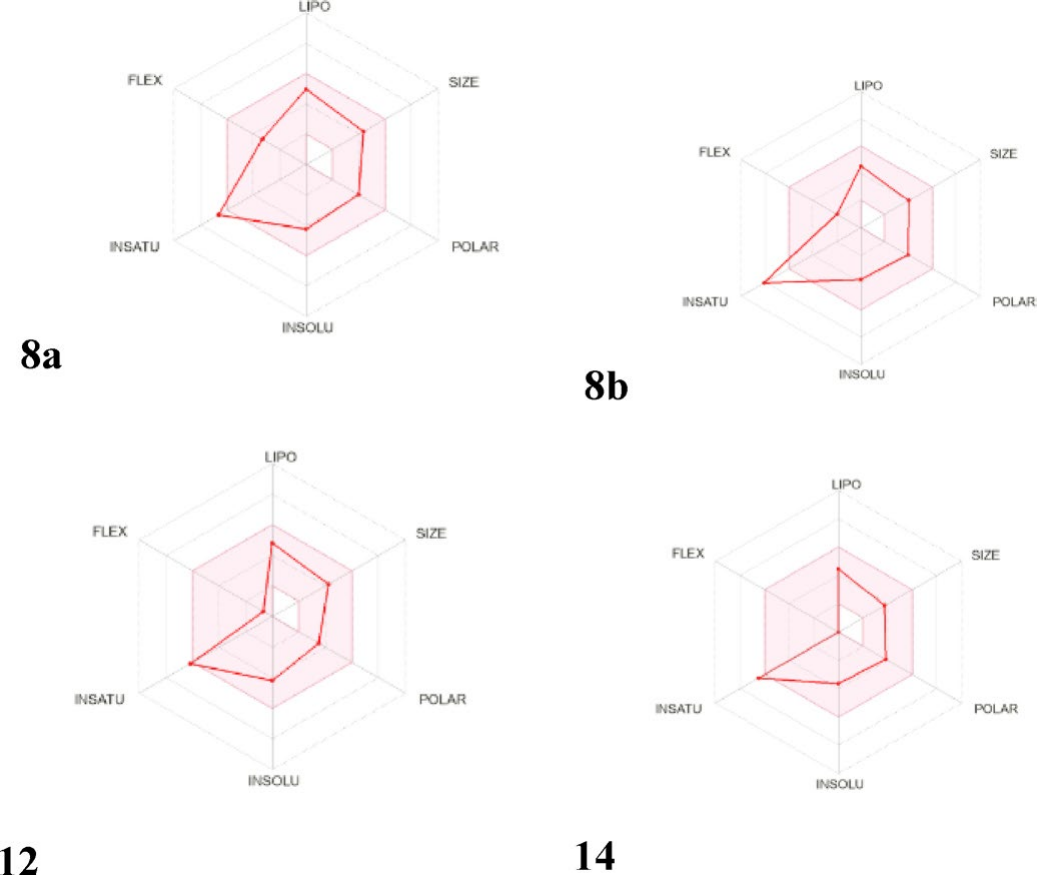
Diagrams showing how compounds **8a**,** 8b**, **12** and **14** correlated to drugs. The colored area is a good physical and chemical space for oral bioavailability. Lipophilicity (LIPO); molecular weight (SIZE); polarity (POLAR); insolubility (INSOLU); in saturation (INSATU); flexibility (FLEX).

For a quick determination of the drug-likeness of chemical scaffolds **8a**,** 8b**, **12** and **14**, bioavailability radar is the most valuable analysis. The bioavailability radar was developed utilising six physiological parameters: size, polarity, solubility, lipophilicity, flexibility and saturation. The BAR prediction showed that the synthesized compounds located within the recommended pink zone (Fig. 14).

**Fig. 15:**
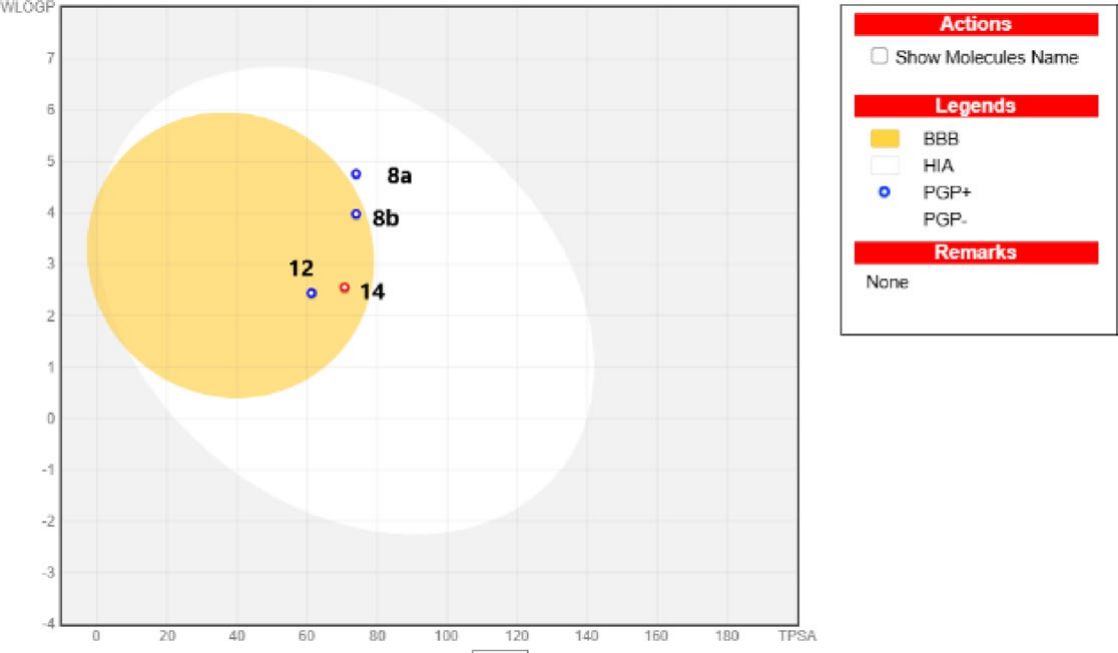
BOILED-EGG diagram of tested compounds **8a**,** 8b**, **12** and **14**.

The BOILED-Egg model illustrated WLOGP vs. TPSA for candidate molecules. The compounds **8b**, **12** and **14** fall within the yellow yolk region indicates potential for good absorption through the gastrointestinal system and capable of crossing the blood–brain barrier (BBB), making thempromising biologically active candidates, except for **8a** which lies in the acceptable HIA region (Fig. 15). In this study, both bioavailability radar and BOILED-Egg analysis were conducted via SwissADME.

The predicted pharmacokinetic properties of compounds **8a**,** 8b**,** 12** and **14** were evaluated using the Swiss ADME web server. As shown in Table 4, all compounds exhibited high gastrointestinal (GI) absorption, indicating favourable oral absorption potential. Compounds **8b**,** 12**, and **14** were predicted to permeate the blood–brain barrier (BBB), whereas compound 8a was predicted to be non-BBB permeant. Compounds **8a**,** 8b**, and **12** were predicted to be P-glycoprotein substrates, while compound **14** was not. The predicted cytochrome P450 inhibition profiles varied among the investigated compounds, suggesting differences in their metabolic behaviour and potential drug–drug interaction profiles. Overall, these in silico pharmacokinetic predictions support the drug-like characteristics of the selected compounds and provide preliminary insight into their ADME properties.

**Table 4:** Predicted Pharmacokinetic Properties of compounds **8a**, **8b**, **12** and **14** using SwissADME.

Compounds	GI absorption	BBB Permeability	*P*-gp substrate	CYP1A2	CYP2C19	CYP2C9	CYP2D6	CYP3A4
**8a**	High	No	Yes	Yes	Yes	Yes	Yes	Yes
**8b**	High	Yes	Yes	Yes	No	Yes	Yes	Yes
**12**	High	Yes	Yes	Yes	No	Yes	Yes	No
**14**	High	Yes	No	No	Yes	No	No	Yes

### Structure activity relationship (SAR) studies

Ten derivatives of substituted indenoquinoxaline hybrids **3**,** 4**,** 8a**,** 8b**,** 10*E***, **11**,** 12**,** 13**,** 14** and **15** were synthesized and assessed against human Caucasian breast adenocarcinoma (MCF7) and human HCT116 [Colon cell line]. Some derivatives showed activity potential with IC50 values ranging from 18.9 to 70.2 µg/mL in human Caucasian breast adenocarcinoma (MCF7), compared to the control drug (Doxorubicin 45.02 µg/mL) and the human HCT116 (Colon cell line) IC50 ranging from 21.4 to 72.9 µg/mL compared to the control drug (Doxorubicin 65.10 µg/mL) (Table 1). Structure-activity relationship (SAR) study was performed on all synthesized compounds, which mainly relied on the characteristics, number, position, steric hindrance and electron-donating/withdrawing effect of the substituents. Herein, the phosphonate derivatives **8a**,** b** exhibited the highest cytotoxic activity among all the screened compounds with promising selectivity against MCF7 with an IC50 value 18.9 and 33.0 µg/mL compared to HCT116 with an IC50 value 52.8 and 72.9 µg/mL, respectively. Conversely, a notable enhancement in activity was observed for compound **8a**, which had a quinoxaline and diethyl phosphonate, compared to the dimethyl phosphonate **8b** against MCF7. This compound contains an ethoxy group which may be contributing to its increased activity compared to the methoxy group. Compound **4**, which has a methyl group, displayed weak activity against MCF7 with an IC50 value 70.2 µg/mL compared to HCT116 with an IC50 value 57.2 µg/mL. Compounds **12** and **14**, which contain pyrazole ring, exhibited moderate activity with IC50 values of 58.6 and 60.9 µg/mL against MCF7 compared to HCT116 with IC50 values of 47.3 and 21.4 µg/mL. Based on these results, The electron-donating group substantially influenced the suppression of activities. However, a subsequent reduction in activity potential was noted in compound **15**, which contains an ethyl group (IC50 = 65.0 and 57.0 µg/mL) compared to compound **14** which, contains methyl group on the pyrazole ring (IC50 = 60.9 and 47.3 µg/mL) against MCF7 and HCT116, respectively.

## Experimental

### Materials and methods

Solvents were purified and desiccated following standard protocols 11*H*-indeno[1,2-*b*]quinoxalin-11-one **(1).**^43^. The reactions were observed, and the purity of the isolated products was assessed using TLC Merck 0.2 mm silica gel 60 F154 analytical aluminium plates. The spots were visualized using UV light at 254 nm and 360 nm. Column chromatography (CC) was performed on silica gel (Kiesel gel 60 mesh, particle size 0.2–0.5 mm; E. Merck, Darmstadt).

Melting points were measured using a Stuart SMP 30 digital melting point apparatus and are reported as uncorrected. IR spectra were recorded on a JASCO FTIR 6100 using KBr disc (JASCO, Japan).

NMR spectra were acquired on a JEOL E.C.A-500 MHz (^13^C: 125.4 MHz, ^1^H: 500.7 MHz, ^31^P: 200.7 MHz) spectrometer (JEOL, Japan) or on a Varian Mercury VX-300 (at 300 MHz) spectrometer using tetramethyl silane (TMS) as an internal reference. ^31^P NMR spectra was obtained using H_3_PO_4_ (85%) as external reference. ^1^H and ^13^C NMR spectra were obtained in deuterated chloroform (CDCl_3_) or in deuterated dimethyl sulfoxide (DMSO-d_6_) with chemical shifts (δ) reported in ppm. Mass spectra (EI-MS) were acquired at 70 eV using a Finnigan MAT SSQ 7000 spectrometer. Elemental analytical data were acquired at the analytical laboratory of the National Research Centre. The cytotoxic activity test [In vitro-bioassay on human tumour cell lines (and) was done and assessed by the Bioassay-Cell Culture Laboratory, National Research Centre, El-Tahrir St., Dokki, Cairo 12622, Egypt.

#### *N’*-(11*H*-indeno[1,2-*b*]quinoxalin-11-ylidene)acetohydrazide (2)

A mixture of 11-hydrazineylidene-11*H*-indeno[1,2-*b*]quinoxaline **(1)** (0.48 g, 2 mmol) with acetic anhydride (10 mL) and acetic acid (5 mL). was subjected to gentle heating under reflux for 2 h (TLC controlled). On completion of reaction the product was separated as white product This product was subjected to filtration, desiccation and recrystallization from ethanol to yield *N’*-(11*H*-indeno[1,2-*b*]quinoxalin-11-ylidene)acetohydrazide **(2).** White crystals, yield (86%), m p 226–228 °C; IR (KBr), *ν* (cm^− 1^): 3210 (NH), 1706 (COCH_3_), 1650, 1612 (C = N, C = C); ^1^H NMR (500 MHz, DMSO d_6_): δ = 2.18 (s, 3 H, COCH_3_), 7.32–7.86 (m, 8 Ar H), 11.90 (s, 1 H, NH) ppm; ^13^C NMR (125 MHz, DMSO d_6_): δ 24.0 (COMe), 116.5, 122.7, 124.9, 127.3, 128.9, 129.2, 129.8, 129.9, 130.3, 131.3, 131.7, 131.8, 132.6, 136.9, 152.1, 155.8 ppm; MS (EI, 70 eV) m/z (%): 288 (11, M^+^), 245 (58), 217 (100), 190 (52), 163 (19), 114 (14), 89 (33), 74 (24); Anal. Calcd. For C_17_H_12_N_4_O: (288.31): C, 70.82; H, 4.20; N, 19.43; Found: C, 70.95; H, 4.32; N, 19.30%.

#### *N’*-(11*H*-Indeno[1,2-*b*]quinoxalin-11-ylidene)-*N*-methylacetohydrazide (3)

To stirred solution of compound **2** (0.56 g, 2 mmol) in acetone (10 mL) using potassium carbonate (1 g), to the mixture, methyl iodide (5 ml) was added. The solution was subjected to reflux at 50 °C for approximately 1 hour. (TLC controlled). The inorganic substance was removed using filtration, and the solution was subsequently evaporated. and concentrated then recrystallized from acetone to give **3.** Pale yellow crystals with yield (83%), mp 201–203 °C; IR (KBr), *ν* (cm^− 1^): 1688 (CO), 1607, 1592 (C = N, C = C); ^1^H NMR (500 MHz, DMSO d_6_): δ = 2.44, 3.80 (2s, 6 H, COCH_3_, NCH_3_), 7.62–8.11 (m, 8 Ar H) ppm; ^13^C NMR (125 MHz, DMSO d_6_): δ 32.7 (Me), 118.2, 119.3, 121.6, 122.6, 129.9, 130.5, 132.2, 137.8, 141.5, 145.2, 155.3, 155.4 ppm ; MS (EI, 70 eV) m/z (%): 302 (67, M^+^), 259 (53), 230 (100), 189 (57), 137 (83), 103(40), 76 (37); Anal. Calcd. For C_18_H_14_N_4_O: (302.34): C, 71.51; H, 4.67; N, 18.53; Found: C, 71.65; H, 4.55; N, 18.67%.

#### 11-(2-Methylhydrazineylidene)-11*H*-indeno[1,2-*b*]quinoxaline (4)

A mixture of compound **3** (0.60 g, 2 mmol) and hydrazine hydrate (5 mL) in absolute ethanol (30 mL) was subjected to reflux heating for 2 h. The progress of reaction was monitored by TLC the solution was evaporated, concentrated and then crystallized from acetone to give **4.**

Deep yellow crystals with yield 75%, mp 118–120 °C; IR (KBr), *ν* (cm^− 1^): 3232 (NH), 1607, 1582 (C = N, C = C); ^1^H NMR (500 MHz, CDCl_3_): *δ* = 3.56 (s, 3 H, NCH_3_), 7.41–8.33 (m, 8 Ar H), 10.85 (s, 1 H, NH) ppm; ^13^C NMR (125 MHz, CDCl_3_): δ 38.2 (Me), 123.4, 126.5, 128.1, 129.1, 129.3, 129.5, 129.7, 130.6, 139.5, 142.2, 145 and 155.6 ppm; MS (EI, 70 eV) m/z (%) 260 (45, M^+^), 231 (100), 216 (35), 156 (11), 138 (12), 89 (33); Anal. Calcd. For C_16_H_12_N_4_: (260.30): C, 73.83; H, 4.65; N, 21.52; Found: C, 73.95; H, 4.54; N, 21.58%.

#### 11-(2,2-Dimethylhydrazineylidene)-11*H*-indeno[1,2-*b*]quinoxaline (5)

A combination of **4** (0.52 g, 2 mmol) and methyl iodide (5 mL) was dissolved in acetone (20 mL) in the presence of potassium carbonate (2 g). The reaction mixture was heated under reflux at 50 °C for about 1 h. (TLC controlled). After the filtration of the inorganic material, the solution was evaporated and concentrated then recrystallized from acetone to give **5.**

Green crystals with yield 80%, mp 125–127 °C; IR (KBr), *ν* (cm^− 1^): 1607, 1582 (C = N, C = C); ^1^H NMR (500 MHz, CDCl_3_): *δ* = 3.12 [s, 6 H, N(CH_3_)_2_], 7.58–8.43 (m, 8 Ar H) ppm; ^13^C NMR (125 MHz, CDCl_3_): δ 46.3 (Me), 123.2, 126.2, 128.0, 129.2, 129.4, 129.7, 129.9, 131.6, 139.3, 142.3, 144.6 and 152.6 ppm; MS (EI, 70 eV) m/z (%) 274 (45, M^+^), 246 (52), 231 (100), 216 (25), 156 (45), 138 (22), 89 (43); Anal. Calcd. For C_17_H_14_N_4_: (274.33): C, 74.43; H, 5.14; N, 20.42; Found: C, 74.51; H, 5.23; N, 20.33%.

#### Reaction of 11-hydrazineylidene-11*H*-indeno[1,2-*b*]quinoxaline (1) and *N’*-(11*H*-indeno[1,2-*b*]quinoxalin-11-ylidene)acetohydrazide (2) with dialkyl phosphites (6a, b)

The preparation of compounds diethyl (10*H*-indeno[1,2-*b*]quinoxalin-11-yl)phosphonate **(8a)** and dimethyl (10*H*-indeno[1,2-*b*]quinoxalin-11-yl)phosphonate **(8b)** had been previously described^54^.

#### Reaction of 11-hydrazineylidene-11*H*-indeno[1,2-*b*]quinoxaline (1) with salicylaldehyde (9)

To a stirred solution of 11-hydrazineylidene-11*H*-indeno[1,2-*b*]quinoxaline **(1)** (0.49 g, 2 mmol) in absolute ethanol (30 mL) and acetic acid (5 mL) the salicylaldehyde **(9)** (0.24 g, 2 mmol) was added. The reaction mixture was heated under reflux for 4 h (TLC controlled). The orange precipitate was collected by filtration and recrystallized from absolute ethanol to afford compounds Schiff base adduct **10** ***E***,*** Z***. Recrystallization of the mixture with acetic acid led to the formation of **10*E*** as pure isomer but the second isomer **10Z** cannot isolated with column or plate chromatography and only identified by ^1^H NMR from the mixture.

#### 2-((*Z*)-(((*E*)-11*H*-Indeno[1,2-*b*]quinoxalin-11ylidene)hydrazineylidene)methyl)phenol (10 *E*)

Orange crystals with yield 80%, mp 177–179 °C; IR (KBr, *ν*_max_) 3443 (OH), 1627 (C = C), 1541 (C = N) cm^− 1^; ^1^H NMR (500 MHz, DMSO-*d*_*6*_): δ 7.00-7.87 (m, 12 H, Ar H), 9.14 (s, 1 H, =CH), 12.33 (s, 1H, OH) ppm; ^13^C NMR (125 MHz, DMSO-*d*_*6*_): δ 117.7, 123.9, 124.2, 126.2, 128.3, 129.2, 129.4, 129.6, 129.7, 129.8, 129.9, 131.2, 138.1, 139.5, 141.5, 142.4, 145.2, 169.1 and 169.6 ppm; EI-MS, m/z (%) 350.38 (M^+^, 40%); Anal. calcd for C_22_H_14_N_4_O; C, 75.42; H, 4.03; N, 15.99; found C, 75.51; H, 4.11; N, 16.08%.

#### 2-((*Z*)-(((*Z*)-11*H*-Indeno[1,2-*b*]quinoxalin-11-ylidene)hydrazineylidene)methyl)phenol (10 *Z*)

^1^H NMR (500 MHz, DMSO-*d*_*6*_): δ 7.12–8.58 (m, 12 H, Ar H), 9.03 (s, =CH), 10.63 (s, OH) ppm.

#### Reaction of 2-((*E*)-(((*Z*)-11*H*-Indeno[1,2-*b*]quinoxaline-11-ylidene)hydrazineylidene) methyl)phenol (10 *E*) with acetic anhydride in acetic acid

A mixture of 2-((*E*)-(((*Z*)-11*H*-Indeno[1,2-*b*]quinoxaline-11-ylidene)hydrazineylidene) methyl)phenol **(10** ***E*)** (0.35 g, 1 mmol) with acetic anhydride (10 mL) and acetic acid (5 mL). was refluxed gently for 1 h (TLC controlled). After the reaction was finished, the product was separated as white product which was filtered, dried and recrystallized from ethanol to give **11.**

#### 2-((*E*)-(((*Z*)-11*H*-Indeno[1,2-*b*]quinoxalin-11-ylidene)hydrazineylidene)methyl)phenyl acetate (11)

White crystals with yield 90%, mp 222–224 °C; IR (KBr, *ν*_max_) 1705 (C = O), 1612 (C = C), 1596 (C = N) cm^− 1^; ^1^H NMR (500 MHz, DMSO-*d*_*6*_): δ 2.32 (s, 3 H, COMe), 7.52 (s, 1 H, =CH), 7.78-8.00 (m, 12 H, Ar H) ppm; ^13^C NMR (125 MHz, DMSO-*d*_*6*_): δ 22.1 (COMe), 101.7, 120.6, 121.3, 122.3, 128.0, 129.4, 130.4, 131.3, 131.6, 132.4, 132.9, 135.4, 139.7, 141.3 and 169.6 ppm; EI-MS, m/z (%) 392.42 (M^+^, 63%); Anal. calcd for C_24_H_16_N_4_O_2_; C, 73.46; H, 4.11; N, 14.28; found C, 73.53; H, 4.20; N, 14.35%.

#### Reaction of 11-hydrazineylidene-11*H*-indeno[1,2-*b*]quinoxaline (1) with ketones in acidic medium

### General procedure

A combination of 11-hydrazineylidene-11*H*-indeno[1,2-*b*]quinoxaline **(1)** (0.48 g, 2 mmol) was dissolved in round bottom flask by 15 mLof dry acetone and/or 2-butanone, then 3 mL of glacial acetic acid was added. Refluxing of the reaction mixture was carried out for about 3 h (TLC controlled). The volatile substances were evaporated to dryness, after which the residue was purified by column chromatography using n-hexane/ethyl acetate to yield **12** and **13.**

#### 2-(5’-Methylspiro[indeno[1,2-*b*]quinoxaline-11,3’-pyrazol]-1‘(2’H)-yl)propan-2-ol (12)

Using *n*-hexane/ethyl acetate (70:30) gave yellow crystals with yield 65%, mp 190–192 °C; IR (KBr, cm^− 1^): *ν* 3361 (OH), 3152 (NH), 1616, 1586 (C = C, C = N) cm^− 1^; ^1^H NMR (500 MHz, CDCl_3_): *δ* 1.58 (2s, 6 H, 2 CH_3_), 2.18 (s, 2 H, CH_2_), 2.95 (s, H, OH), 7.42–8.20 (m, 8 ArH, ), 11.15 (s, 1H, NH) ppm; ^13^C NMR (125 MHz, CDCl_3_): δ 19.1 (Me), 25.6 (Me), 80.2, 94.5, 98.1, 122.6, 127.2, 128.3, 128.5, 129.7, 129.9, 130.0, 130.6, 132.3, 140.3, 140.7, 142.3, 144.5, 145.3, 157.6 and 162.6 ppm; EI MS m/z (%) 344 (M^+^, 100%); Analysis calculated for C_21_H_20_N_4_O (344.42): C, 73.23; H, 5.85; N, 16.27; Found: C, 73.32; H, 5.91; N, 16.38%.

#### 4-Ethyl-5,6-dimethyl-1,7-diazaspiro[bicyclo[2.2.1]heptane-2,11’-indeno[1,2-b]quinoxalin]-6-ol (13)

Using *n-*hexane/ethyl acetate (80:20) gave orange crystals with yield 70%, mp 102–104 °C; IR (KBr, cm^− 1^): *ν* 3351 (OH), 3155 (NH), 1610 (C = N) cm^− 1^; ^1^H NMR (500 MHz, CDCl_3_): *δ* 1.01 (t, *J*_*HH*_ = 7.2 Hz, 3 H, -CH_2_**CH**_**3**_), 1.12 (d, *J*_*HH*_ = 7.2 Hz, 3 H, -CH**CH**_**3**_), 1.53 (s, 3 H, CH_3_), 1.97 (q, *J*_*HH*_ = 7.2 Hz, 1 H, -**CH**CH_3_), 2.49 (q, *J*_*HH*_ = 7.2 Hz, 2 H, -**CH**_**2**_CH_3_), 2.85, 3.00 (2d, *J*_*HH*_ = 15.3 Hz, 2 H, CH_2_), 7.51–8.16 (m, 8 ArH), 11.16 (s, 1 H, NH) ppm; ^13^C NMR (125 MHz, CDCl_3_): δ 2.4 (CH**CH**_**3**_), 7.5 (-CH_2_**CH**_**3**_), 22.3 (CH_3_), 30.1 (-**CH**_**2**_CH_3_), 55.7 (CH_2_), 60.2 (-**CH**CH_3_), 67.8, 80.2, 100.0, 122.1, 126.2, 128.4, 128.6, 128.7, 129.4, 140.3, 142.3 and 158.6 ppm; EI MS m/z (%) 372 (M^+^, 22%); Analysis calculated for C_23_H_24_N_4_O (372.47): C, 74.17; H, 6.49; N, 15.04; Found: C, 74.26; H, 6.60; N, 15.12%.

#### Reaction of 11-hydrazineylidene-11*H*-indeno[1,2-*b*]quinoxaline (1) with ketones in potassium carbonate

### General procedure

A mixture of 11-hydrazineylidene-11*H*-indeno[1,2-*b*]quinoxaline **(1)** (0.48 g, 2 mmol) was dissolved in round bottom flask by 15 mL of dry acetone and/or 2-butanone, then 2 g of potassium carbonate was added. The reaction mixture was maintained under reflux for about 3 h. (TLC controlled). The inorganic material was removed using filtration, and the solvent was subsequently evaporated tell dryness then, the residue was purified by column chromatography using n-hexane/ethyl acetate to give **14** and **15**.

#### 5’-Methyl-4’,5’-dihydrospiro[indeno[1,2-b]quinoxaline-11,3’-pyrazol]-5’-ol (14)

Using n-hexane/ethyl acetate (40:60%) gave off-white crystals with yield 75%, mp up to 300 °C; IR (KBr, cm^− 1^): *ν* 3361 (OH), 1616, 1586 (C = C, C = N) cm^− 1^; ^1^H NMR (500 MHz, CDCl_3_): *δ* 2.15 (s, 3 H, CH_3_), 3.24, 3.94 (2 d, *J*_*HH*_ = 17 Hz, 2 H, CH_2_), 5.19 (s, 1 H, OH), 7.46–8.07 (m, 8 H, ArH) ppm; ^13^C NMR (125 MHz, CDCl_3_): δ 23.2 (Me), 66.5 (CH_2_), 70.2, 85.3, 122.7, 128.2, 129.3, 129.4, 129.7, 130.0, 130.4, 130.6, 132.3, 145.3, 157.6 and 162.6 ppm; EI MS m/z (%) 302 (M^+^, 23%); Analysis calculated for C_18_H_14_N_4_O (302.34): C, 71.51; H, 4.67; N, 18.53; Found: C, 71.60; H, 4.62; N, 18.64%.

#### 5’-Ethyl-4’,5’-dihydrospiro[indeno[1,2-*b*]quinoxaline-11,3’-pyrazol]-5’-ol (15)

Using n-hexane/ethyl acetate (50:50%) produced off-white crystals with yield 68%, mp 214–216 °C; IR (KBr, cm^− 1^): *ν* 3365 (OH), 1614, 1587 (C = C, C = N) cm^− 1^; ^1^H NMR (500 MHz, CDCl_3_): *δ* 1.01 (t, *J*_*HH*_ = 7.2 Hz, 3 H, CH_2_**Me**), 2.41 (s, 1 H, OH), 2.42 (q, *J*_*HH*_ = 7.0 Hz, 2 H, **CH**_**2**_Me), 3.19, 3.42 (2 d, *J*_*HH*_ = 17 Hz, 2 H, CH_2_), 7.46–8.07 (m, 8 H, ArH) ppm; ^13^C NMR (125 MHz, CDCl_3_): *δ* 5.4 (CH_2_**Me**), 31.5 (**CH**_**2**_Me), 67.4 (**CH**_2_), 72.3 (C), 91.4 (**C**OHEt), 119.6, 122.4, 129.0, 129.3, 129.4, 129.7, 129.9, 130.0, 130.6, 132.3, 145.3, 157.6 and 162.6 ppm; EI MS m/z (%) 316 (M^+^, 15%); Analysis calculated for C_19_H_16_N_4_O (316.36): C, 72.13; H, 5.10; N, 17.71; Found: C, 72.22; H, 5.06; N, 17.82%.

#### Reaction of 11-hydrazineylidene-11*H*-indeno[1,2-*b*]quinoxaline (1) with urea in acetic acid

A mixture of **1** (0.25 g, 1 mmole) and urea **(16)** (0.060, 1 mmole) in acetic acid (5 mL) was refluxed for 3 h, and the reaction progress was monitored by TLC. After completion of the reaction, water added, and the resulting precipitate was collected, dried and recrystallized from acetic acid to afford compound **2**, which identified by and mixed melting point with an authentic sample.

### Cytotoxicity screening by MTT assay

Cell viability was assessed by the mitochondrial dependent reduction of yellow MTT (3-(4,5-dimethylthiazol-2-yl)-2,5-diphenyltetrazoliumbromide) to purple formazan^55^ in triplicate.

Procedure: All the following procedures were done in a sterile area using a Laminar flow cabinet biosafety class II level (Baker, SG403INT, Sanford, ME, USA). Cells were suspended in EMEM medium for HePG2, 1% antibiotic-antimycotic mixture (10,000 U/mL Potassium Penicillin, 10,000 µg/mL Streptomycin Sulfate and 25 µg/mL Amphotericin B) and 1% L-glutamine at 37 °C under 5% CO_2_. Cells were cultured for 10 days and then seeded at a density of 1 × 10^4^ cells/well in fresh complete growth medium in 96-well microtiter plastic plates at 37 °C for 24 h under 5% CO_2_ using a water jacketed Carbon dioxide incubator (Sheldon, TC2323, Cornelius, OR, USA). Media was aspirated, fresh medium (without serum) was added and cells were incubated either alone (negative control) or with different concentrations of sample to give a final concentration of (100-50-25-12.5-6.25-3.125-0.78 and 1.56 µg/mL). After 48 h of incubation, medium was aspirated. Forty microlitres of MTT solution (2.5 µg/mL) were added to each well and incubated for further four hours at 37 °C under 5% CO_2_. To stop the reaction and dissolving the formed crystals, 200 µL of 10% Sodium dodecyl sulphate (SDS) in deionized water was added to each well and incubated overnight at 37 °C. A positive control (doxorubicin) consisting of a known cytotoxic agent (100 µg/mL) was included^56,57^.

The absorbance was then measured using a microplate multi-well reader (Bio-Rad Laboratories Inc., model 3350, Hercules, California, USA) at 595 nm and a reference wavelength of 620 nm. A statistical significance was tested between samples and negative control (cells with vehicle) using independent t-test by SPSS 11 program. DMSO is the vehicle used for dissolution of plant extracts and its final concentration on the cells was less than 0.2%. The percentage of change in viability was calculated according to the formula:

$$\left( {\left( {{\text{Reading of extract }}/{\text{ Reading of negative control}}} \right){\text{ }} - {\mathrm{1}}} \right){\text{ }} \times {\text{ 1}}00$$

A probit analysis was performed to determine the IC₅₀ values using SPSS version 11.

In the present study, the degree of selectivity of the synthetic compounds is expressed as: SI = IC_50_ of a pure compound in a normal cell line/IC_50_ of the same pure compound in a cancer cell line, where IC_50_ is the concentration required to kill 50% of the cell population.

#### Expression of *BCl-2*, *BAX* and P53 in MCF-7 cell lines

### RNA isolation and reverse transcription (RT) Reaction

RNeasy Mini Kit (Qiagen, Hilden, Germany) supplemented with DNaseI (Qiagen) digestion step were used to isolate total RNA from negative and treated breast cancer cell lines (MCF-7) according to the manufacturer’s protocol. Isolated total RNA was treated with one unit of RQ1 RNAse-free DNAse (Invitrogen, Germany) to digest DNA residues, re-suspended in DEPC-treated water and quantified photo spectrometrically at 260 nm. Purity of total RNA was assessed by the 260/280 nm ratio which was between 1.8 and 2.1^58^. Additionally, integrity was assured with ethidium bromide-stain analysis of 28 S and 18 S bands by formaldehyde-containing agarose gel electrophoresis. Aliquots were used immediately for reverse transcription (RT), otherwise they were stored at -80 °C.

Complete Poly(A) + RNA isolated from negative and treated MCF-7 cell lines was reverse transcribed into cDNA in a total volume of 20 µl using RevertAid™ First Strand cDNA Synthesis Kit (Fermentas, Germany). An amount of total RNA (5 µg) was used with a master mix. The master mix was consisted of 50 mM MgCl_2_, 10x RT buffer (50 mM KCl; 10 mM Tris-HCl; pH 8.3), 10 mM of each dNTP, 50 µM oligo-dT primer, 20 IU ribonuclease inhibitor (50 kDa recombinant enzyme to inhibit RNase activity) and 50 IU MuLV reverse transcriptase. The mixture of each sample was centrifuged for 30 s at 1000 g and transferred to the thermocycler. The RT reaction was carried out at 25 °C for 10 min, followed by 1 h at 42 °C, and finished with a denaturation step at 99 °C for 5 min. Afterwards the reaction tubes containing RT preparations were flash-cooled in an ice chamber until being used for cDNA amplification through quantitative Real Time- polymerase chain reaction (qRT-PCR).

### Quantitative real time- PCR (qRT-PCR)

Determination the negative and treated MCF-7 cancer cell lines cDNA copy number was carried out using StepOne™ Real-Time PCR System from Applied Biosystems (Thermo Fisher Scientific, Waltham, MA USA). PCR reactions were set up in 25 µL reaction mixtures containing 12.5 µL 1× SYBR**®** Premix Ex TaqTM (TaKaRa, Biotech. Co. Ltd.), 0.5 µL 0.2 µM sense primer, 0.5 µL 0.2 µM antisense primer, 6.5 µL distilled water, and 5 µL of cDNA template. The reaction program was allocated to 3 steps. First step was at 95.0 °C for 3 min. Second step consisted of 40 cycles in which each cycle divided to 3 steps: (a) at 95.0 °C for 15 s; (b) at 55.0 °C for 30 s; and (c) at 72.0 °C for 30 s. The third step consisted of 71 cycles which started at 60.0 °C and then increased about 0.5 °C every 10 s up to 95.0 °C. Each experiment included a distilled water control. The sequences of specific primers of *Bcl-2*^59^; Bax^60^ and P53 genes^61,62^ were designed and listed in Table 5. At the end of each qPCR a melting curve analysis was performed at 95.0 °C to check the quality of the used primers. The relative quantification of the target to the reference was determined by using the 2^*−*ΔΔCT^ method^63,64^.

**Table 5:** Primers sequence used for *qRT-PCR* of MCF-7 cell lines.

Gene	Primer sequence	GenBank (accession no)
*Bcl-2*	F: cct cgc tgc aca aat act cc R: tgg aga gaa tgt tgg cgt ct	NM_000633.3
*BAX*	F: aag aag ctg agc gag tgt ct R: gtt ctg atc agt tcc ggc ac	NM_001291428.2
*P53*	F: tgg cca. tct aca agc agt ca. R: ggt aca gtc aga gcc aac ct	NM_001126112.3
*GAPDH*	F: cac atc gct cag aca cca. tg R: tga cgg tgc cat gga att tg	NM_001289745.3

*BCL2*: BCL2 apoptosis regulator, *BAX*: BCL2 associated X, apoptosis regulator; *GAPDH*: Glyceraldehyde-3-phosphate dehydrogenase.

### Cell culture & sample preparation

#### Procedure

Cells were collected from each well using Trypsin and then centrifuged for 5 min at 1500 rpm. Cell pellets were washed with 1 mL DPBS and collected by centrifugation as above. Cells were fixed using 70% ethanol on ice for 1 h. The ethanol-suspended cells were centrifuged for 5 min at 300 ×g and ethanol decanted thoroughly. The cell pellets were re-suspended in 1 mL 1x DPBS for 15 min and was centrifuged at 300 ×g for 5 min. Cell pellets were resuspended in RNAse solution for 30 min at 37 °C. The cell pellets were re-suspended in 200 µl of Propedium iodide solution and kept in the dark at 4 °C s for 30 min. Cells were then transferred to the CytoFLEX Flow Cytometer (Beckman Coulter Life Sciences, USA) to measure the cell fluorescence (Beckman Coulter Inc., Cairo, Egypt and Cat. No. 4238055-CB) for cell cycle analysis. The percentage of cells in G0/G1, S, and G2/M phases of the cell cycle was calculated using CytExpert Software.

### Molecular docking

#### Protein preparation for molecular docking

The protein preparation was executed via PyMOL. The protein structures were sourced from the RCSB-PDB database and prepped for docking by eliminating water molecules.

These compounds were docked into several proteins linked to breast cancer (PDB ID: 6vj3) and colon cancer (PDB ID: 6GUE) for protein 6GUE water molecules and all protein chains except chain A were deleted prior to docking. Using PyRx software, the protein structure was prepared as a PDBQT file, with hydrogens assigned to all polar residues. The docking studies were conducted using AutoDock Vina, positioning the grid boxes over the functional domains of each receptor. The specific grid center coordinates applied during the docking were: PDB ID: 6vj3 X: 11.823 Y: -3.07 Z: 15.936 and PDB ID: 6GUE X: -8.916 Y: -21.72 Z: 9.67. The best conformation was chosen with the lowest docked energy, following completion of the docking procedure, the conformation exhibiting the lowest docked energy was chosen. For each ligand, ten docking runs were conducted using AutoDock Vina, and the optimal pose from each run was retained. The mean affinity of these best poses was taken as the final binding energy.

#### Ligand preparation for molecular docking

Using ChemDraw Ultra 2008, 2D ligand structures were sketched, minimized with Chem3D Ultra, and saved as PDB files. The four structures of **8a**, **8b**, **12** and **14** were computationally arranged and orientated within the binding site of the target protein. Diverse scoring systems and methods were utilized to assess the binding affinity and calculate the binding free energy between the ligands and the protein. The docking scores, measured in kcal/mol, quantify the binding energy between each compound and the target protein a more negative docking score indicates a stronger binding interaction between the compound and the protein.

### Swiss ADME analysis

The compounds’ physicochemical characteristics covering lipophilicity, aqueous solubility, pharmacokinetics, drug-likeness, medicinal chemistry features, and bioavailability were computed using Swiss ADME (http://www.swissadme.ch). Bioavailability offers a rapid evaluation of the potential for oral uptake of compounds to ascertain their appropriateness as oralmedications. Utilizing Swiss ADME, the physicochemical and ADME characteristics of these four compounds can be shown. SwissADME evaluates the risk involved in giving people or other animals a medicinal drug.

## Conclusion

In summary, a series of new indenoquinoxaline derivatives was synthesized, were characterized and assessed for their anticancer activity. The ten compounds demonstrated significant inhibition of proliferation in MCF-7 and HCT-116 cell lines. Specifically, phosphonate derivatives **8a** and **8b** showed the strongest cytotoxicity toward MCF-7 cells, while compounds **12** and **14** were the most potent against HCT-116 cells. In addition, some compounds exhibited moderate to inactive cytotoxicity. The SAR investigation has been performed on the desired compounds and elucidated that the type of the substituent on the indenoquinoxaline moiety affects their antitumor activities.

Molecular docking analysis showed that **8a** had higher binding energy (-9.4 kcal/mol) than other synthesised hybrids against breast protein (6vj3), while compound **14** had higher binding energy (-8.8 kcal/mol) against colon protein (6GUE).

## Supplementary Information

Below is the link to the electronic supplementary material.

Supplementary Material 1

Supplementary Material 2

## Data Availability

The authors declare that the data supporting the finding of this study are available in supplementary files.
