# Supplementary material for: Synthesis of novel 11H-indeno[1,2-b]quinoxaline derivatives and their in vitro and in silico insights into anticancer activities
Source: Sci Rep. 2026 Aug 12;16:25135. doi: 10.1038/s41598-026-64175-7 (PMC13469509; doi:10.1038/s41598-026-64175-7)
Supplement: Supplementary file 1 [file 41598_2026_64175_MOESM1_ESM.docx]

**Supporting Information**

**Synthesis of novel** **11*H*-indeno[1,2-*b*]quinoxaline derivatives and there in vitro and in silico insights into anticancer activities**

**Fatma A. El-Samahy^*a^, Ehab M. Zayed^a^, Ghada A. Eldeken^a^, Fayez H. Osman^a^, Khaled Mahmoud^b^ and Galal E. H. Elgemeie^c^**

**Contents**

**I. Figures**

a. Some biologically active indeno[1,2-*b*]quinoxaline derivative. **Figure 1**........................................ **S1**
b. The structure of compound **8a,b** in the crystal**. Figure 2**................................................................... **S2**
c. Crystal structure of proteins (PDB ID: 6GUE chain A) and (PDB ID: 6VJ3). **Figure 3** ..........…..... **S3**
d. Molecular docking of compounds **8a** (A, B**)**, **8b** (C, D), **12** (E, F) and **14** (G, H) with breast protein
e. (6vj3) 3D and 2D. **Figure 4** ............. ……………………………………………….…………….... **S4**
f. Molecular docking of compounds **8a** (A, B**)**, **8b** (C, D), **12** (E, F) and **14** (G, H) with colon protein (6GUE) 3 D and 2 D. **Figure 5**.......................................................................................................... **S5**
g. Diagrams showing how compounds **8a, 8b**, **12** and **14** correlated to drugs. The colored area is a good physical and chemical space for oral bioavailability. Lipophilicity (LIPO); molecular weight (SIZE); polarity (POLAR); insolubility (INSOLU); in saturation (INSATU); flexibility (FLEX). **Figure 6**.......................................... **S6**
h. BOILED-EGG diagram of tested compounds **8a, 8b**, **12** and **14**. **Figure 7**...................................... **S7**

**II. Schemes**

a. Synthesis of 11-(hydrazineylidene)-11*H*-indeno[1,2-*b*]quinoxaline derivatives. **Scheme1**……............. **S8**
b. The reaction of dialkyl phosphites with indenoquinoxaline derivatives. **Scheme 2**………….………..… **S9**
c. Synthesis of (((Z)-11*H*-indeno[1,2-*b*]quinoxalin-11ylidene)hydrazineylidene)methyl)phenol (**10 E, Z**). **Scheme 3 S10**
d. The reaction of 11-hydrazineylidene-11*H*-indeno[1,2-*b*]quinoxaline with carbonyl compounds in presence of acetic acid **Scheme 4** …...............................................................................................................................................................**S11**
e. The reaction of 11-hydrazineylidene-11*H*-indeno[1,2-*b*]quinoxaline with carbonyl compounds in presence of potassium carbonate. **Scheme 5** ………………………………………………..………**S12**
f. Reaction of hydrazone **1** with urea. **Scheme 6** …………………… …………………………….…. **S13**

**III. Tables.**

a. Cytotoxity of the synthesized 11H-indeno[1,2-b]quinoxalin-11-one derivatives towards human Caucasian breast adenocarcinoma (MCF7) and human HCT116 [Colon cell line]. **Table 1**....................................... **S14**
b. Docking energy (kcal/mol) and binding interactions of the docked ligands into the active MAPK8

human proteins. **Table 2**................................................................................................................. **S15**

c. The physicochemical parameters of the compounds **8a**, **8b**, **12** and **14. Table 3**................................ **S16**

**IV. Spectra.........................................................................................................................................** .... **S17-S46**

**S1**

**8a	8b**

**S2**

**S3**

| **A**   | **B**   |
| --- | --- |
| **C**   | **D**   |
| **E**   | **F**   |
| **G**   | **H**   |

**S4**

| **A**   | **B**   |
| --- | --- |
| **C**   | **D**   |
| **E**   | **F**   |
| **G**   | **H**   |

**S5**

| **8a**  | **8b**  |
| --- | --- |
|   **12** |   **14** |

**S6**

**S7**

**S8**

**S9**

**S****10**

**S11**

**S12**

**S13**

| **Sample Code** | **IC_50_ of MCF-7**  **Cells (µM)** | **IC_50_ of HCT116**  **Cells (µM)** | **Sample Code** | **IC_50_ of MCF-7**  **Cells (µM)** | **IC_50_** **of (HCT116**  **Cells (µM)** |
| --- | --- | --- | --- | --- | --- |
| **3** | 60.0 | ND^[a]^ | **12** | 58.6 | 47.3 |
| **4** | 70.2 | 57.2 | **13** | ND | ND |
| **8a** | 18.9 | 52.8 | **14** | 65 | 21.4 |
| **8b** | 33.0 | 72.9 | **15** | 60.9 | 57 |
| **10 *E*** | ND | ND | **DMSO** | ND | ND |
| **11** | ND | ND | **Negative control** | ND | ND |

**S14**

| **Compounds** | **Receptor** | **Conventional Hydrogen bond** | **Pi Donor**  **Hydrogen bond** | **Pi-alkyl/Alkyl** | **Pi-Pi Tshaped /**  **Pi-Pi T staacked** | **Pi- Sigma** | **Pi- cation**  **/pi-anion** | **Binding energy**  **(Kcal/mol)** |
| --- | --- | --- | --- | --- | --- | --- | --- | --- |
| **8a** | 6vj3 | ASN62(2.13Å) | THR199(3.36Å) | LEU198(4.7 Å)  VAL143(6.06 Å)  VAL121(5.2 Å,4.9 Å  HIS96(4.45 Å)  ALA65(3.59 Å) |  |  |  | -9.4 |
| **8b** | 6vj3 | GLN92(2.36 Å)  ASN67(2.18Å) |  | VAL121(4.78 Å)  VAL143(4.79 Å)  LEU198(3.8 Å)  ALA65(4.7 Å) |  | LEU198(3.82 Å)  THR200(3.80 Å) |  | -8.6 |
| **12** | 6vj3 | PRO201 (2.42 Å) |  | VAL121 (4.69 Å) | PHE131  (4.90Å)  HIS94  (5.25Å) | LEU198 (3.84 Å) |  | -8.2 |
| **14** | 6vj3 | ASN 67(2.77 Å)  ASN62(2.60 Å)  (2.29 Å) |  | LEU198(5.01 Å) |  | HIS94(3.97 Å) |  | -8 |
| **8a** | 6GUE | LYS129(2.45Å) (2.57Å) | TYR15(3.22Å) | TYR15(4.76Å) |  | VAL18(3.92Å) | ASP86(3.75Å) | -7.2 |
| **8b** | 6GUE | TYR15(2.97 Å)  LYS129(2.67Å) |  | VAL18(5.07 Å) |  | VAL 18(3.80 Å) | ASP 86(3.79 Å) | -7 |
| **12** | 6GUE | LYS 250  (5.33 Å)  GLN 246  (5.16 Å) |  | ARG214  (5.74 Å)  ARG217  (6.50 Å)  LEU202  (5.82 Å) |  |  |  | -7.7 |
| **14** | 6GUE | GLU 12  (2.76 Å)  TYR15  (1.85 Å) |  | VAL18  (4.99Å) |  | VAL 18  (3. 65 Å) | ASP86  (3.56 Å) | -8.8 |

**S15**

| **Compounds** | **MW** | **Rotatable bonds** | **H-bond acceptors** | **H- bond**  **donors** | **TPSA** | **(I LOG P)** | **Lipinski violation** | **Bioavailability Score** |
| --- | --- | --- | --- | --- | --- | --- | --- | --- |
| **8a** | 354.34 g/mol | 5 | 4 | 1 | 74.02 Å² | 3.18 | 0 | 0.55 |
| **8b** | 326.29 g/mol | 3 | 4 | 1 | 74.02 Å² | 2.88 | 0 | 0.55 |
| **12** | 344.41 g/mo | 1 | 4 | 2 | 61.28 Å² | 2.99 | 0 | 0.55 |
| **14** | 302.33 g/mol | 0 | 5 | 1 | 70.73Å² | 2.64 | 0 | 0.55 |

**S16**

**(^1^H NMR of 2) S17**

**(IR of 2) S18**

**(**MS of 2**) S19**

**(^1^H NMR of 3) S20**

**(MS of 3) S21**

**(IR of 3) S22**

**(^1^H NMR of 4) S23**

**(MS of 4) S24**

**(IR of 4) S25**

**(^1^H NMR of 8a) S26**

**(^13^C NMR of 8a) S27**

**(31P NMR of 8a) S28**

**(MS of 8a) S29**

**(IR of 8a) S30**

**(**^1^H NMR of **8b) S31**

**(**^31^P NMR of **8b) S32**

**(**MS of **8b) S33**

(IR of **8b) S34**

**(**^1^H NMR of **10*E*) S35**

**(**^1^H NMR of **10*E*** and **10*Z*) S36**

(MS of **10*E*) S37**

(IR of **10*E***) **S38**

**(**^1^H NMR of **12**) **S39**

(MS of **12**) **S40**

(IR of **12**) **S41**

**(**^1^H NMR of **13**) **S42**

(MS of **13**) **S43**

(IR of **13**) **S44**

**(**^1^H NMR of **13**) **S45**

(MS of **13**) **S46**
