# Supplementary material for: Synthesis of novel 11H-indeno[1,2-b]quinoxaline derivatives and their in vitro and in silico insights into anticancer activities
Source: Sci Rep. 2026 Aug 12;16:25135. doi: 10.1038/s41598-026-64175-7 (PMC13469509; doi:10.1038/s41598-026-64175-7)
Supplement: Supplementary file 2 [file 41598_2026_64175_MOESM2_ESM.docx]

**Supporting Information**

**Synthesis of novel** **11*H*-indeno[1,2-*b*]quinoxaline derivatives and there in vitro and in silico insights into anticancer activities**

**Fatma A. El-Samahy^*a^, Ehab M. Zayed^a^, Ghada A. Eldeken^a^, Fayez H. Osman^a^, Khaled Mahmoud^b^ and Galal E. H. Elgemeie^c^**

**Contents**

**I. Figures**

a. Some biologically active indeno[1,2-*b*]quinoxaline derivative. **Figure 1**............................................ **S1**
b. The structure of compound **8a,b** in the crystal**. Figure 2**...................................................................... **S2**
c. The alterations of *Bcl-2* gene expression in negative and treated MCF-7 cell lines. Data are

presented as mean ± SEM. ^a,b,c:^ Mean values within tissue with unlike superscript

letters were significantly different (*P*<0.05). **Figure 3 …………………………………….….…..... S3**

d. The alterations of *BAX* gene expression in negative and treated MCF-7 cell lines.

Data are presented as mean ± SEM. ^a,b,c:^ Mean values within tissue with unlike superscript letters

were significantly different (*P*<0.05). **Figure4………………………………………………………S4**

e. The alterations of *P53* gene expression in negative and treated MCF-7 cell lines.

Data are presented as mean ± SEM. ^a,b,c:^ Mean values within tissue with unlike

superscript letters were significantly different (*P*<0.05). **Figure 5………………………………..…S5**

f. Crystal structure of proteins (PDB ID: 6GUE chain A) and (PDB ID: 6VJ3). **Figure 6** ..........…..... **S6**
g. Molecular docking of compounds **8a** (A, B), **8b** (C, D), **12** (E, F) and **14** (G, H) with breast protein (6vj3) 3 D and 2 D. **Figure 7** .......…………………………………………….……………….….... **S7**
h. Molecular docking of compounds **8a** (A, B**)**, **8b** (C, D), **12** (E, F) and **14** (G, H) with

colon protein (6GUE) 3 D and 2 D. **Figure 8**...................................................................................... **S8**

i. Molecular docking of co-crystallized 6GUE (A, B) and co-crystalized 6VJ3 (C, D)

**Figure 9**................................................................................................................................................ **S9**

j. Rmsd of co-crystalized 6VJ3 and co-crystallized 6GUE. **Figure 10**................................................. **S10**
k. Diagrams showing how compounds **8a, 8b**, **12** and **14** correlated to drugs. The colored area

is a good physical and chemical space for oral bioavailability. Lipophilicity (LIPO);

molecular weight (SIZE); polarity (POLAR); insolubility (INSOLU); in saturation

(INSATU); flexibility (FLEX). **Figure 11**....................................................................................... **S11**

l. BOILED-EGG diagram of tested compounds **8a, 8b**, **12** and **14**. **Figure 12**.................................... **S12**

**II. Schemes**

a. Synthesis of 11-(hydrazineylidene)-11*H*-indeno[1,2-*b*]quinoxaline derivatives. **Scheme1**……......... **S13**
b. The reaction of dialkyl phosphites with indenoquinoxaline derivatives. **Scheme 2**………….…… .… **S14**
c. Synthesis of (((Z)-11*H*-indeno[1,2-*b*]quinoxalin-11ylidene)hydrazineylidene)methyl)phenol (**10 E, Z**).

**Scheme 3** ………………………………………………………………………………………………**. S15**

d. The reaction of 11-hydrazineylidene-11*H*-indeno[1,2-*b*]quinoxaline with carbonyl compounds in presence

of acetic acid **Scheme 4**…….................................................................................................................................**S16**

e. The reaction of 11-hydrazineylidene-11*H*-indeno[1,2-*b*]quinoxaline with carbonyl

compounds in presence of potassium carbonate. **Scheme 5** ………………………………..……………**S17**

**III. Tables.**

a. Cytotoxity of the synthesized 11H-indeno[1,2-b]quinoxalin-11-one derivatives towards human

Caucasian	breast adenocarcinoma (MCF7) and human HCT116 [Colon cell line]. **Table1**..................... **S18**

b. Docking energy (kcal/mol) and binding interactions of the docked ligands into the active

MAPK8 human proteins. **Table 2**........................................................................................................ **S19**

c. The physicochemical parameters of the compounds **8a**, **8b**, **12** and **14. Table 3**................................. **S20**
d. Primers sequence used for qRT-PCR of MCF-7 cell lines. **Table 4…………………….……………. S21**

**IV. Spectra**...................................................................................................................................... ……... **S22-S54**

**S 1**

**8a	8b**

**S 2**

| Treatment | Relative expression of Bcl-2 (Bcl-2/GAPDH) |
| --- | --- |
| MCF-7 (-ve) | 1 |
| MCF-7+ (+ve) | 0.39 |
| MCF-7+T(G) | 0.68 |

| **Treatment** | **Mean** | **SEM** | **(% of control)** |
| --- | --- | --- | --- |
| MCF-7 (-ve) | 1.00 | 0.056 | 100.0 |
| MCF-7+ (+ve) | 0.39 | 0.034 | 39.0 |
| MCF-7+T(G) | 0.68 | 0.048 | 68.0 |

**S 3**

| Treatment | Relative expression of BAX (BAX/ GAPDH) |
| --- | --- |
| MCF-7 (-ve) | 1 |
| MCF-7+ (+ve) | 6.78 |
| MCF-7+T(G) | 4.2 |

| **Treatment** | **Mean** | **SEM** | **(% of control)** |
| --- | --- | --- | --- |
| MCF-7 (-ve) | 1.00 | 0.135 | 100.0 |
| MCF-7+ (+ve) | 6.78 | 0.351 | 678.0 |
| MCF-7+T(G) | 4.20 | 0.263 | 420.0 |

**S 4**

| Treatment | Relative expression of P53 (P53/ GAPDH) |
| --- | --- |
| MCF-7 (-ve) | 1 |
| MCF-7+ (+ve) | 8.66 |
| MCF-7+T(G) | 5.32 |

| **Treatment** | **Mean** | **SEM** | **(% of control)** |
| --- | --- | --- | --- |
| MCF-7 (-ve) | 1.00 | 0.184 | 100.0 |
| MCF-7+ (+ve) | 8.66 | 0.470 | 866.0 |
| MCF-7+T(G) | 5.32 | 0.250 | 532.0 |

**S 5**

**S 6**

| **A**   | **B**   |
| --- | --- |
| **C**   | **D**   |
| **E**   | **F**   |
| **G**   | **H**   |

**S 7**

| **A**   | **B**   |
| --- | --- |
| **C**   | **D**   |
| **E**   | **F**   |
| **G**   | **H**   |

**S 8**

| **A**   | **B**   |
| --- | --- |
| **C**   | **D**   |

**S 9**

|  |  |
| --- | --- |

**S 10**

| **8a**  | **8b**  |
| --- | --- |
|   **12** |   **14** |

**S 11**

**S 12**

**S 13**

**S 14**

**S** **15**

**S 16**

**S 17**

| **Sample Code** | **IC_50_ of MCF-7**  **Cells (µM)** | **IC_50_ of HCT116**  **Cells (µM)** | **Sample Code** | **IC_50_ of MCF-7**  **Cells (µM)** | **IC_50_** **of (HCT116**  **Cells (µM)** |
| --- | --- | --- | --- | --- | --- |
| **3** | 60.0±1.5 | ND^[a]^ | **12** | 58.6±2.3 | 47.3±1.4 |
| **4** | 70.2±1.6 | 57.2±0.8 | **13** | ND | ND |
| **8a** | 18.9±1.2 | 52.8±0.9 | **14** | 65±1.7 | 21.4±0.9 |
| **8b** | 33.0±1.2 | 72.9±0.6 | **15** | 60.9±0.3 | 57±0.8 |
| **10 *E*** | ND | ND | **DMSO** | ND | ND |
| **11** | ND | ND | **Negative control** | ND | ND |
| **+ve control**  **Doxorubicin** | 45.02±1.3 | 65.1±0.9 |  |  |  |

[a] ND, not detected **±S.D**

**S 18**

| **Compounds** | **Receptor** | **Conventional Hydrogen bond** | **Pi Donor**  **Hydrogen bond** | **Pi-alkyl/Alkyl** | **Pi-Pi Tshaped /**  **Pi-Pi T staacked** | **Pi- Sigma** | **Pi- cation**  **/pi-anion** | **Binding energy**  **(Kcal/mol)** |
| --- | --- | --- | --- | --- | --- | --- | --- | --- |
| **8a** | 6vj3 | ASN62(2.13Å) | THR199(3.36Å) | LEU198(4.7 Å)  VAL143(6.06 Å)  VAL121(5.2 Å,4.9 Å  HIS96(4.45 Å)  ALA65(3.59 Å) |  |  |  | -9.4 |
| **8b** | 6vj3 | GLN92(2.36 Å)  ASN67(2.18Å) |  | VAL121(4.78 Å)  VAL143(4.79 Å)  LEU198(3.8 Å)  ALA65(4.7 Å) |  | LEU198(3.82 Å)  THR200(3.80 Å) |  | -8.6 |
| **12** | 6vj3 | PRO201 (2.42 Å) |  | VAL121 (4.69 Å) | PHE131  (4.90Å)  HIS94  (5.25Å) | LEU198 (3.84 Å) |  | -8.2 |
| **14** | 6vj3 | ASN 67(2.77 Å)  ASN62(2.60 Å)  (2.29 Å) |  | LEU198(5.01 Å) |  | HIS94(3.97 Å) |  | -8 |
| **8a** | 6GUE | LYS129(2.45Å) (2.57Å) | TYR15(3.22Å) | TYR15(4.76Å) |  | VAL18(3.92Å) | ASP86(3.75Å) | -7.2 |
| **8b** | 6GUE | TYR15(2.97 Å)  LYS129(2.67Å) |  | VAL18(5.07 Å) |  | VAL 18(3.80 Å) | ASP 86(3.79 Å) | -7 |
| **12** | 6GUE | LYS 250  (5.33 Å)  GLN 246  (5.16 Å) |  | ARG214  (5.74 Å)  ARG217  (6.50 Å)  LEU202  (5.82 Å) |  |  |  | -7.7 |
| **14** | 6GUE | GLU 12  (2.76 Å)  TYR15  (1.85 Å) |  | VAL18  (4.99Å) |  | VAL 18  (3. 65 Å) | ASP86  (3.56 Å) | -8.8 |

**S 19**

| **Compounds** | **MW** | **Rotatable bonds** | **H-bond acceptors** | **H- bond**  **donors** | **TPSA** | **(I LOG P)** | **Lipinski violation** | **Bioavailability Score** |
| --- | --- | --- | --- | --- | --- | --- | --- | --- |
| **8a** | 354.34 g/mol | 5 | 4 | 1 | 74.02 Å² | 3.18 | 0 | 0.55 |
| **8b** | 326.29 g/mol | 3 | 4 | 1 | 74.02 Å² | 2.88 | 0 | 0.55 |
| **12** | 344.41 g/mo | 1 | 4 | 2 | 61.28 Å² | 2.99 | 0 | 0.55 |
| **14** | 302.33 g/mol | 0 | 5 | 1 | 70.73Å² | 2.64 | 0 | 0.55 |

**S 20**

| **Gene** | **Primer sequence** | **GenBank**  **(accession no)** |
| --- | --- | --- |
| ***Bcl-2*** | F: cct cgc tgc aca aat act cc  R: tgg aga gaa tgt tgg cgt ct | NM_000633.3 |
| ***BAX*** | F: aag aag ctg agc gag tgt ct  R: gtt ctg atc agt tcc ggc ac | NM_001291428.2 |
| ***P53*** | F: tgg cca tct aca agc agt ca  R: ggt aca gtc aga gcc aac ct | NM_001126112.3 |
| ***GAPDH*** | F: cac atc gct cag aca cca tg  R: tga cgg tgc cat gga att tg | NM_001289745.3 |

**S 21**

**(^1^H NMR of 2) S 22**

**(^13^C NMR of 2) S 23**

**(IR of 2) S 24**

**(**MS of **2) S 25**

**(^1^H NMR of 3) S 26**

**(^13^C NMR of 3) S 27**

**(MS of 3) S 28**

**(IR of 3) S 29**

**(^1^H NMR of 4) S 30**

**(MS of 4) S 31**

**(IR of 4) S 32**

**(^1^H NMR of 8a) S 33**

**(^13^C NMR of 8a) S 34**

**(31P NMR of 8a) S 35**

**(MS of 8a) S 36**

**(IR of 8a) S 37**

**(**^1^H NMR of **8b) S 38**

**(**^31^P NMR of **8b)** **S 39**

**(^13^C NMR of 8b) S 40**

**(**MS of **8b) S 41**

(IR of **8b) S 42**

**(**^1^H NMR of **10*E*) S 43**

**(**^1^H NMR of **10*E*** and **10*Z*) S 44**

(MS of **10*E*) S 45**

(IR of **10*E***) **S 46**

**(**^1^H NMR of **12**) **S 47**

(MS of **12**) **S 48**

(IR of **12**) **S 49**

**(**^1^H NMR of **13**) **S 50**

(MS of **13**) **S 51**

(IR of **13**) **S 52**

**(**^1^H NMR of **13**) **S 53**

(MS of **13**) **S 54**
